# Quality Measures to Enhance the Management and Treatment of Primary Biliary Cholangitis: A Delphi Consensus Study

**DOI:** 10.1111/liv.70118

**Published:** 2025-05-02

**Authors:** Domenico Alvaro, Vincenza Calvaruso, Marco Carbone, Nora Cazzagon, Annarosa Floreani, Pietro Invernizzi, Marco Marzioni, Patrizio Pasqualetti, Grazia Pennisi, Pierluigi Toniutto, Umberto Vespasiani‐Gentilucci, Antonio Craxì

**Affiliations:** ^1^ Sapienza University of Rome Rome Italy; ^2^ Department of Health Promotion, Mother and Child Care, Internal Medicine and Medical Specialties (PROMISE), Section of Gastroenterology and Hepatology University of Palermo Palermo Italy; ^3^ Department of Medicine and Surgery, Gastroenterology University of Milano‐Bicocca Milan Italy; ^4^ Department of Surgery, Oncology and Gastroenterology University of Padua Padua Italy; ^5^ University of Padua Padua Italy; ^6^ Scientific Consultant of IRCSS (Scientific Institute for Research, Hospitalization and Healthcare) Verona Italy; ^7^ Division of Gastroenterology, Centre for Autoimmune Liver Diseases, European Reference Network on Hepatological Diseases (ERN RARE‐LIVER) IRCCS Fondazione San Gerardo Dei Tintori Monza Italy; ^8^ Department of Medicine and Surgery University of Milano‐Bicocca Monza Italy; ^9^ Clinic of Gastroenterology AOU Ospedali Riuniti, Polytechnic University of Marche Ancona Italy; ^10^ Department of Public Health and Infectious Diseases, Section of Health Statistics and Biometry Faculty of Pharmacy and Medicine, Sapienza University of Rome Rome Italy; ^11^ Hepatology and Liver Transplant Unit University of Udine Udine Italy; ^12^ Unità di Epatologia Università Campus Biomedico di Roma Rome Italy; ^13^ Unità di Medicina Clinica Ed Epatologia Fondazione Policlinico Universitario Campus Bio‐Medico di Roma Rome Italy

**Keywords:** Delphi consensus methodology, non‐invasive diagnostic tools, obeticholic acid (OCA), primary biliary cholangitis (PBC), quality measures in PBC management

## Abstract

**Background & Aims:**

Primary Biliary Cholangitis (PBC) is a chronic autoimmune liver disease characterised by bile duct destruction, leading to cholestasis and fibrosis. Despite therapeutic advancements, gaps remain in diagnostic standardisation, treatment response evaluation, and patient‐centred care. This study aimed to develop consensus‐driven quality measures to optimise PBC management.

**Methods:**

Using the Delphi methodology, 92 clinicians participated in two rounds of surveys addressing diagnostic protocols, therapeutic strategies, follow‐up standards, and patient‐reported outcomes (PROs). Appropriateness ratings were analysed using the RAND/UCLA method to achieve consensus on key quality measures.

**Results:**

Strong consensus was reached on the appropriateness of non‐invasive diagnostic tools such as Vibration‐Controlled Transient Elastography (VCTE) and abdominal ultrasound for staging and monitoring fibrosis in chronic cholestasis. Different treatment options were evaluated in patients with inadequate response to UDCA, including compensated cirrhosis (Child‐Pugh A), with early initiation deemed appropriate in cases of UDCA intolerance or partial response. Genetic studies and liver biopsy showed variability in consensus, particularly for patients without biochemical cholestasis, reflecting areas needing further research.

**Conclusions:**

This study establishes actionable quality measures for PBC care, offering specific recommendations on diagnostic protocols, therapeutic benchmarks, and follow‐up standards. These measures go beyond guidelines by addressing gaps in patient stratification, follow‐up protocols, and strategies. Future research should address cost‐effectiveness, access to non‐invasive tools, and implementation challenges such as clinician training and resource availability.

**Summary**

- This study used the Delphi methodology to develop shared quality measures to optimise the management of primary biliary cholangitis (PBC).
- A strong consensus was reached on the appropriateness of non‐invasive diagnostic tools such as vibration‐controlled transient elastography (VCTE) and abdominal ultrasound for staging and monitoring fibrosis in chronic cholestasis.
- The study defines feasible quality measures for the treatment of PBC, providing specific recommendations on diagnostic protocols, treatment parameters, and follow‐up standards.
- Future research should focus on cost‐effectiveness, access to non‐invasive tools, and implementation challenges such as training of physicians and availability of resources.

## 1 Introduction

Primary Biliary Cholangitis (PBC) is a chronic autoimmune liver disease marked by the destruction of interlobular bile ducts, leading to cholestasis, fibrosis, and, if untreated, cirrhosis [1, 2]. Its aetiology involves genetic predisposition and environmental triggers, initiating an autoimmune response targeting small bile ducts. Antimitochondrial antibodies (AMAs) are present in 90%–95% of patients, alongside elevated alkaline phosphatase (ALP), key biomarkers of disease activity [1, 3]. Predominantly affecting women (10:1 ratio), diagnosis peaks between ages 40–60 and is more prevalent in Northern Europe and North America [4, 5]. UDCA improves survival, but 20%–40% of patients require second‐line therapies like obeticholic acid or PPAR agonists, the latter being used off‐label [3, 6].

Despite therapeutic advances, PBC significantly impacts patients' quality of life, often due to persistent symptoms such as fatigue and pruritus. This underscores the need for a patient‐centred approach that integrates clinical efficacy with quality‐of‐life considerations. Current clinical guidelines emphasise pharmacological treatments but often lack protocols addressing patient‐reported outcomes (PROs) and standardised diagnostic tools, which this study aims to refine. Additionally, the absence of streamlined diagnostic protocols contributes to variability in disease staging and monitoring, increasing the potential for delayed or inconsistent care. Furthermore, significant heterogeneity in clinical management has been observed across different regions, reflecting disparities in access to diagnostic tools, therapeutic options, and follow‐up protocols. These gaps highlight the need for a standardised, consensus‐driven approach to optimise care for PBC patients. Notably, there is a lack of studies that critically evaluate existing guidelines to identify potential gaps, underscoring the importance of further research to refine recommendations and address unmet clinical needs in PBC care.

The Delphi methodology is particularly suited to address these challenges, as it allows for the integration of expert clinical input to develop quality measures that bridge the gaps in current guidelines. By focusing on tangible, actionable recommendations, this study aims to standardise care processes, enhance diagnostic efficiency, and improve patient‐centred outcomes in PBC management.

In this study, expert consensus highlighted the importance of Vibration‐Controlled Transient Elastography (VCTE) as a non‐invasive and reliable tool for assessing liver stiffness and staging fibrosis in patients with PBC. Additionally, there was strong agreement to offer, as long as it was possible, the obeticholic acid (OCA) as a second‐line treatment for patients with an inadequate response to UDCA, emphasising its role in delaying disease progression and improving transplant‐free survival. It is made clear that in November 2024, the EC's decision to rule out granting approval for obeticholic acid as a treatment for PBC became effective. However, the purpose of all these tangible recommendations is to provide a general framework for optimising diagnostic and therapeutic strategies in PBC care.

This project aimed to develop consensus‐driven quality measures to enhance the diagnosis, management, and treatment of PBC. Quality measures are standardised benchmarks that assess the appropriateness, effectiveness, and efficiency of healthcare interventions. In this study, these measures were developed using the Delphi methodology, which incorporates structured expert consensus to identify key areas of improvement in PBC care. The developed quality measures include:

1. Diagnostic Standards: Ensuring the use of validated biomarkers (e.g., ALP, bilirubin, GGT) and non‐invasive techniques (e.g., elastography) for accurate disease staging and monitoring.
2. Therapeutic Benchmarks: Defining criteria for the initiation and optimisation of first‐ and second‐line treatments, such as UDCA, OCA, and combination therapies.
3. Follow‐Up Protocols: Establishing evidence‐based intervals for treatment response assessments and long‐term monitoring.
4. Patient‐Centred Care Indicators: Addressing symptom management (e.g., pruritus) and incorporating patient‐reported outcomes into routine care.

These measures go beyond existing clinical guidelines by providing a framework to standardise care delivery and evaluate healthcare performance across diverse clinical settings. While clinical guidelines primarily focus on evidence‐based recommendations, the quality measures developed in this project emphasise actionable standards for evaluating care processes and outcomes, facilitating their implementation in routine practice.

## 2 Methods

### 2.1 Delphi Methodology

The Delphi methodology was employed to achieve consensus among experts in hepatology, gastroenterology, and internal medicine, with a focus on diagnostic tools, treatment strategies, and follow up. Originally developed by the RAND Corporation, this well‐established method fosters consensus‐building among experts [7, 8, 9, 10]. Based on core principles such as anonymity, controlled feedback, and statistical group response, the Delphi method is extensively used in health research to address clinical challenges [11].

### 2.2 Development of the Delphi

**FIGURE 1:**
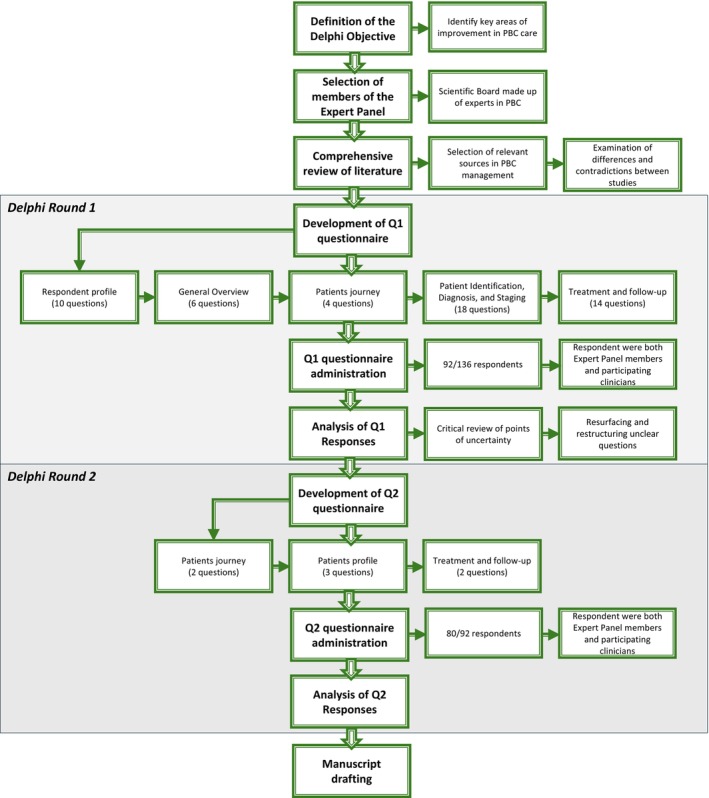
Delphi study flow chart.

The development of the Delphi survey followed a multi‐stage process (Figure 1), starting with a comprehensive review of the literature to identify gaps and guide the formulation of the initial questionnaire (Q1). The literature review focused on key topics in the management of primary biliary cholangitis (PBC), including diagnostic protocols, treatment strategies, and follow‐up practices.

This study, conducted between February and July 2024, was led by a scientific board composed of experts recognised for their expertise in PBC. Following a comprehensive literature review, a Delphi survey was developed, consisting of two questionnaires (Q1 and Q2), which were subsequently distributed to an Expert Panel (EP) of participating clinicians. The literature search incorporated specific MeSH terms to ensure a targeted and systematic review of evidence, including: (“Primary Biliary Cholangitis/diagnosis”[MeSH Terms] AND “Biomarkers/blood”[MeSH Terms] AND “Liver Function Tests”[MeSH Terms]); (“Primary Biliary Cholangitis/therapy”[MeSH Terms] AND “Obeticholic Acid/therapeutic use”[MeSH Terms] AND “Treatment Outcome”[MeSH Terms]); (“Primary Biliary Cholangitis”[MeSH Terms] AND “Patient Care Management/standards”[MeSH Terms] AND “Follow‐Up Studies”[MeSH Terms]); and (“Delphi Technique”[MeSH Terms] AND “Consensus Development Conferences as Topic”[MeSH Terms] AND “Primary Biliary Cholangitis”[MeSH Terms]).

The structure of Q1 was as follows:

- General Overview (6 questions) and Patient Journey (4 questions) section, designed to map diagnostic resources and facilities available at the respondent's clinical centre and to understand how patient referrals are managed in the respondent's clinical centre;
- Patient identification, diagnosis and staging (18 questions) section, including multiple diagnosis and staging clinical scenarios corresponding to different diagnostic and clinical management of the patient;
- Treatment and Follow‐up (14 questions) section, to explore the use of therapies available at the time of the survey for the treatment of PBC and its short‐ and long‐term complications.

In each question, respondents were asked to indicate the importance or appropriateness of each proposed multiple‐choice answer by assigning a score from 1 (minimal importance/appropriateness) to 9 (maximum importance/appropriateness).

To ensure clarity and minimise ambiguity, the first round of the Delphi survey (Q1) also served as a pilot test. Respondents were encouraged to provide feedback on question wording, relevance, and clarity. The Scientific Board incorporated this feedback to refine the questionnaire and ensure that subsequent iterations were more precise and aligned with the objectives of the study.

**FIGURE 2:**
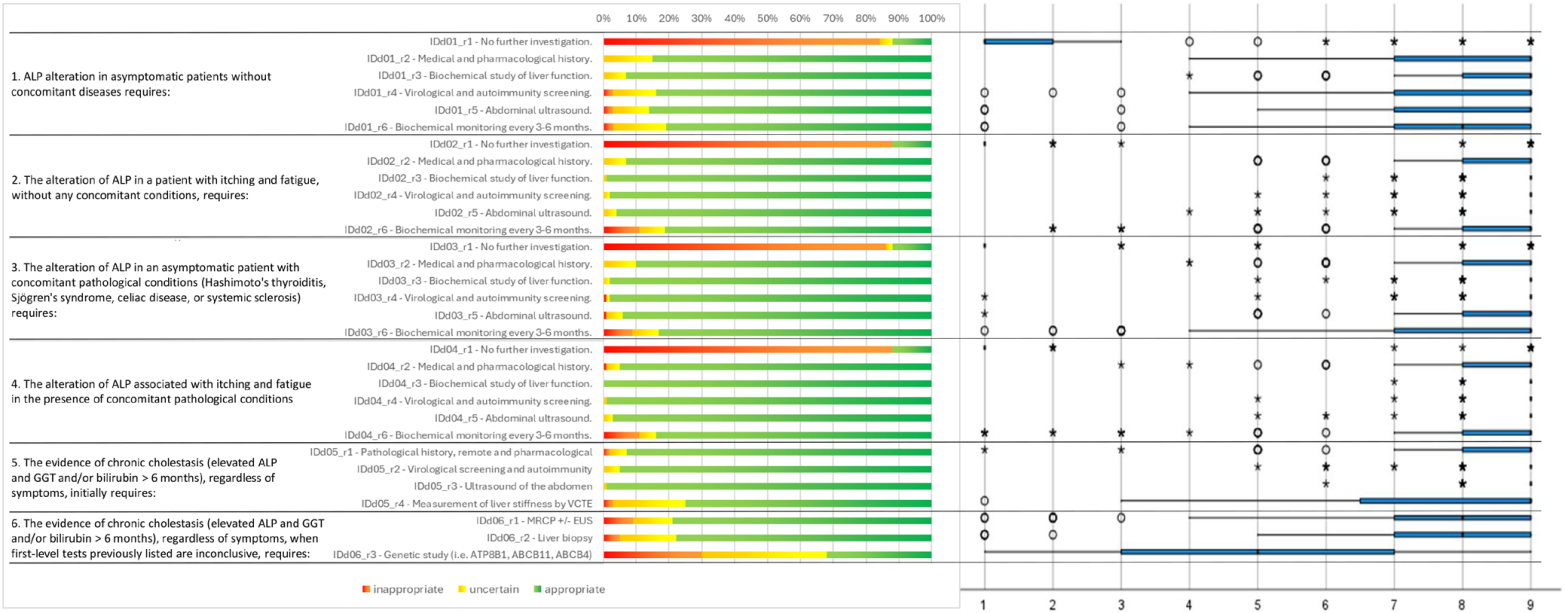
Round 1—identification and diagnosis—questions 1–6.

**FIGURE 3:**
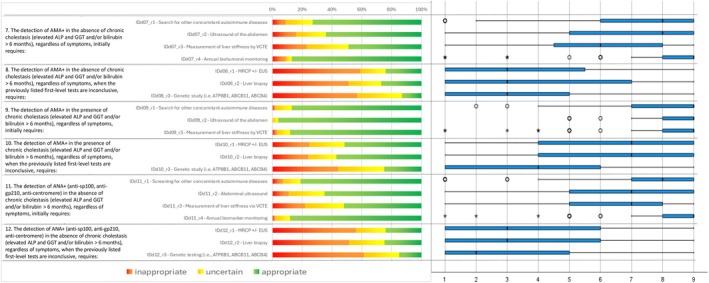
Round 1—identification and diagnosis—questions 7–12.

**FIGURE 4:**
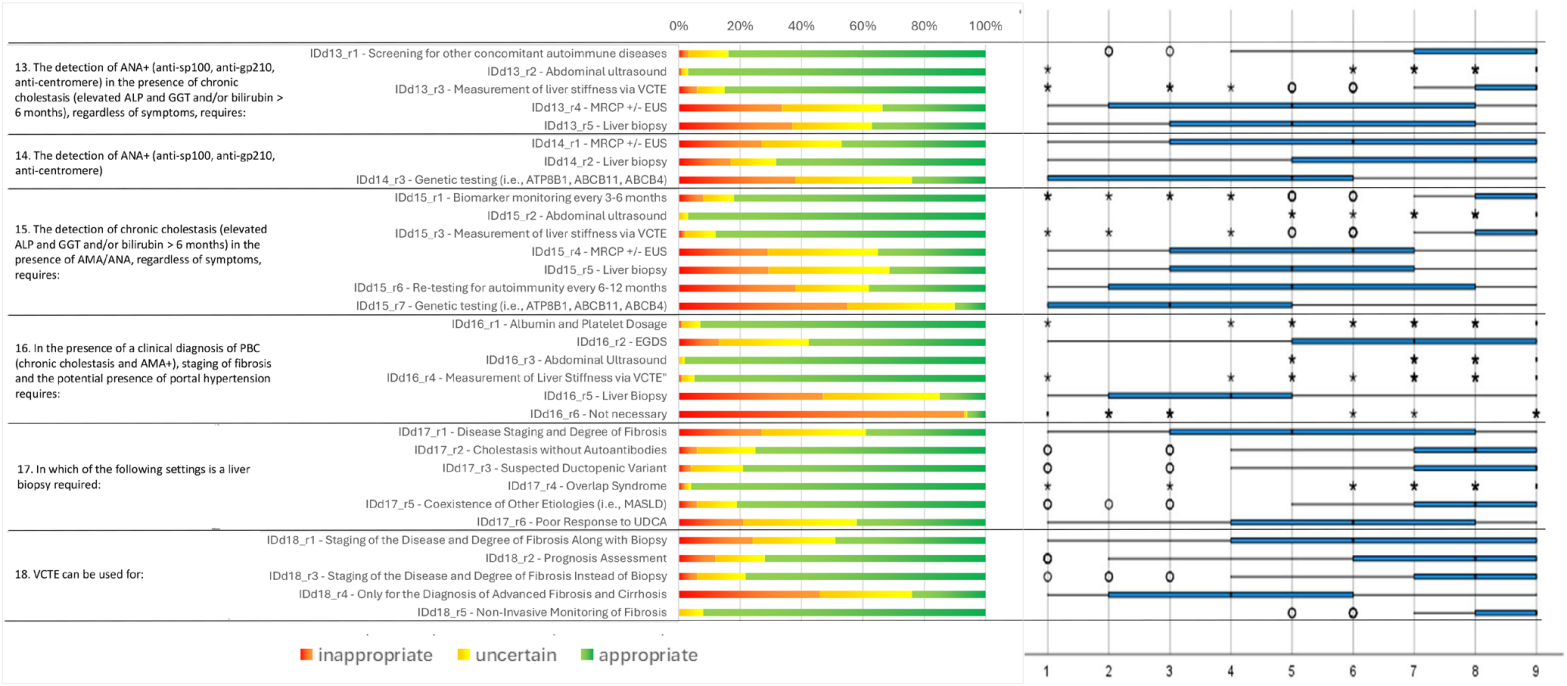
Round 1—identification and diagnosis—questions 13–18.

**FIGURE 5:**
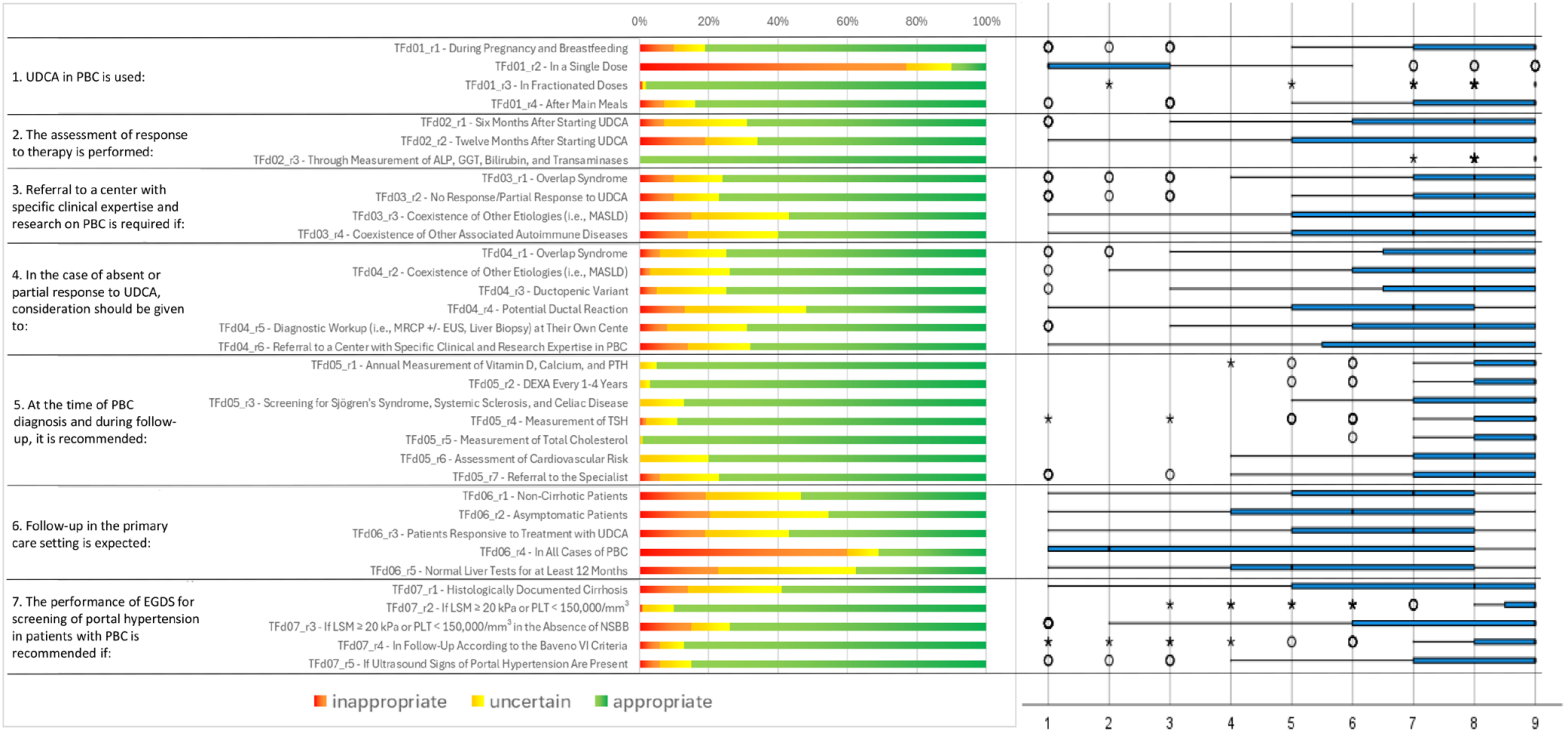
Round 1—treatment and follow‐up—questions 1–7.

**FIGURE 6:**
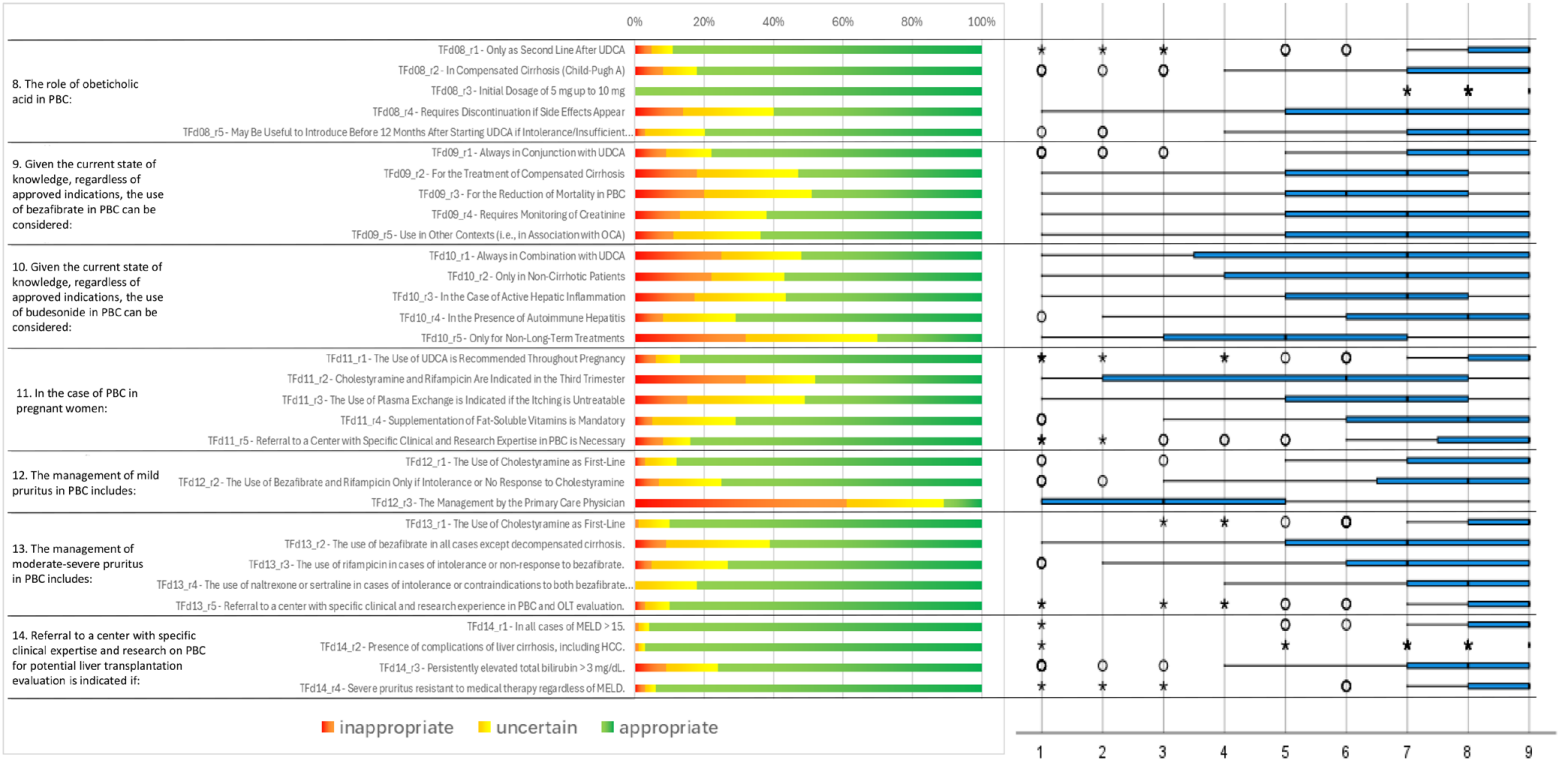
Round 1—treatment and follow‐up—questions 8–14.

**FIGURE 7:**
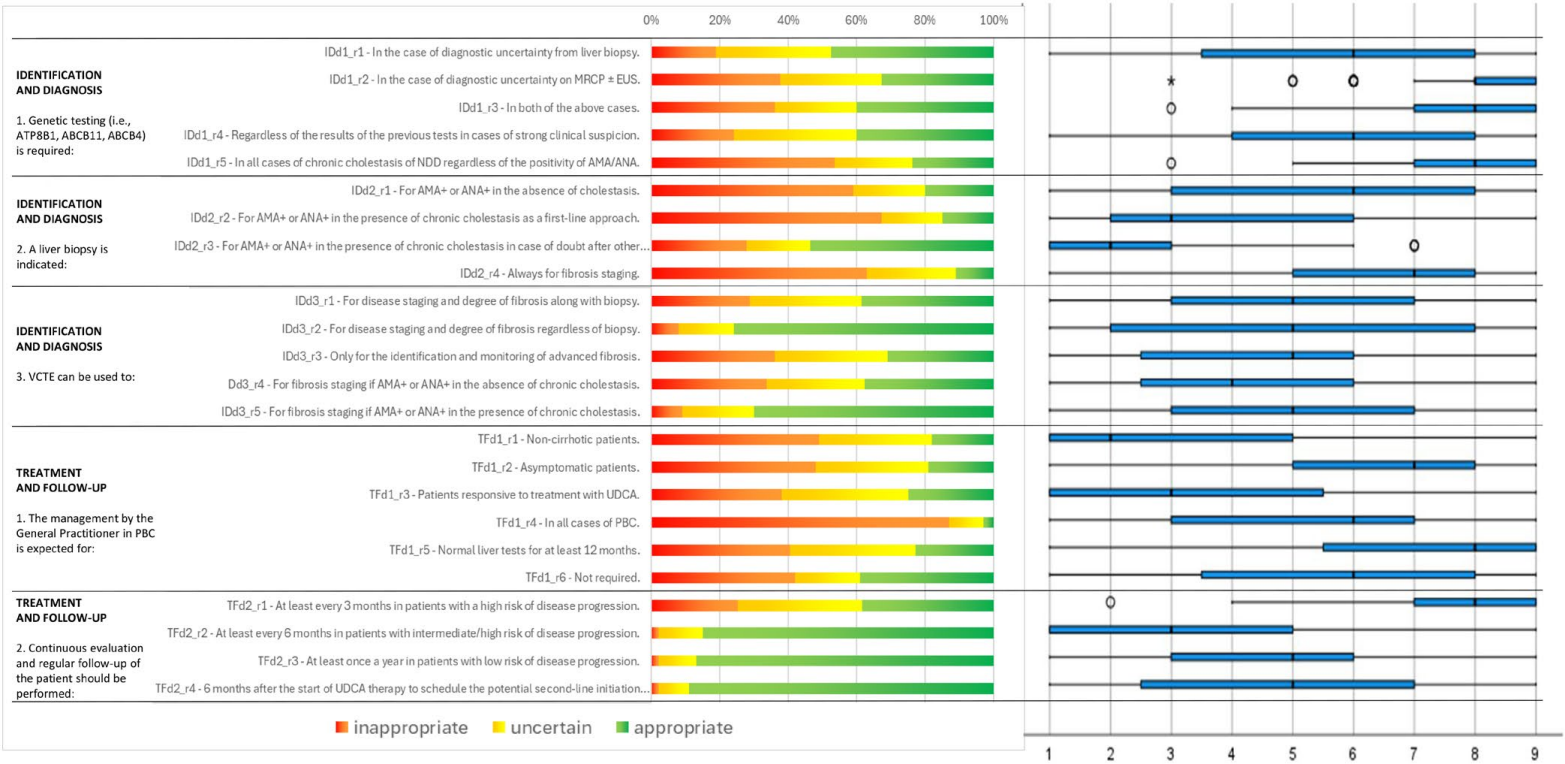
Round 2—identification and diagnosis—questions 1–3/treatment and follow‐up—questions 1–2.

Following the collection and analysis of Q1 responses (Figures 2, 3, 4, 5, 6), the Scientific Board evaluated whether any elements failed to receive unanimous ratings from the responding centres and, consequently, could be addressed in a second questionnaire (Q2) for further investigation. Questions included in Q2 were those where ratings showed significant variability or ambiguity, or where additional clarity was needed to achieve consensus (Figure 7). The Q2 questionnaire included only those questions requiring further clarification, categorised into the following sections: Patient Journey (2 questions), Patient Profile (3 questions), and Treatment and Follow‐up (2 questions). A structured process guided the progression of questions from Q1 to Q2. Items were included, excluded, or reformulated based on predefined criteria, including response dispersion, median scores below the consensus threshold, and qualitative feedback from participants. This iterative process ensured that Q2 focused exclusively on unresolved or contentious topics. The Scientific Board reviewed questionnaires Q1 and Q2 to ensure their scientific content, wording, and overall appropriateness. The final versions of Q1 and Q2 (Data S2) were prepared and made available in a software platform (Sawtooth Software) for real‐time collection of responses from Delphi participants.

### 2.3 Selection of Participants and Distribution of Questionnaires

Initially, the questionnaires were distributed across the Italian territory to an EP consisting of 136 clinicians, selected by the Scientific Board as experts in the pathology under study and members of specialist networks: Italian PBC Registry and/or the Club Epatologi Ospedalieri (CLEO) and/or the Associazione Italiana Gastroenterologi e Endoscopisti Digestivi Ospedalieri (AIGO) PBC Task Force and/or the Sicilian PBC Network and/or the PBC Project Piemonte‐Liguria‐Valle D'Aosta.

Q1 was distributed to the EP via email in February 2024, and statistical analysis was performed on the responses from 92 participants. Following the rules for an effective and rigorous conduct of a Delphi Study, the questionnaire was completely anonymized and none of the respondents knew the answers of the other participating clinicians, ensuring confidentiality and unbiased participation. Based on the responses to Q1, adjustments were made, and Q2 was distributed via email in July 2024 to the 92 clinicians who had participated in the first round. Responses to Q2 were received from 80 experts, after which the data was statistically analysed and compared to the Q1 responses (Data S1).

### 2.4 Statistical Analysis

Respondents' agreement was assessed using the Delphi method, following the guidelines established by the RAND Corporation [12]. This method utilises a scale ranging from 1 (maximum disagreement) to 9 (maximum agreement), with a score of 5 representing a neutral opinion on a specific item. The scores provided by respondents were then statistically analysed to calculate an appropriate “consensus index”. In line with “The RAND/UCLA Appropriateness Method User's Manual”, the Inter‐Percentile Range Adjusted for Symmetry (IPRAS) was used as a measure of score dispersion adjusted for symmetry, to determine the level of agreement for each item. The rationale is that when ratings are symmetrical, the Inter‐Percentile Range (IPR) required to classify an indication as disagreement is smaller compared to when the ratings are asymmetrical. Asymmetry was defined as “the distance between the central point of the IPR and the central point of the 1–9 scale, i.e., 5”. As the asymmetry of ratings increases, a larger IPR is required to indicate disagreement. The following mathematical function was developed: IPRAS = IPRr + (AI × CFA), where IPRr is the IPR required for disagreement under perfect symmetry, AI is the Asymmetry Index, and CFA is the Correction Factor for Asymmetry. The IPRAS threshold is dependent on the symmetry of the ratings around the median, meaning that each item requires its own IPRAS calculation. Consequently, an item is rated as having disagreement if IPRi > IPRASi.

Based on the computation of IPR and IPRAS, each statement can be classified regarding the appropriateness of a given diagnostic or therapeutic strategy into the following categories: Appropriate (panel median of 7–9, without disagreement), Uncertain (panel median of 4–6 or any median with disagreement), and Inappropriate (panel median of 1–3, without disagreement).

According to Italian Law, surveys conducted with healthcare professionals do not require approval from Ethical Committees. Participants who completed the questionnaire explicitly provided their consent to participate in the study.

Data analysis was performed using SPSS 27.0 (IBM), and a scoring sheet was developed in Excel to calculate all necessary statistics as outlined in “The RAND/UCLA Appropriateness Method User's Manual”.

## 3 Results

### 3.1 General Characteristics of the Respondents

Ninety‐two clinicians completed the questionnaires. The median age of the respondents was 55 years (range: 29–69 years). The median experience of the clinicians in managing PBC was 22 years (range: 4–43 years). Physicians were distributed across 18 of the 20 Italian regions, though not evenly (Lombardy, Sicily and Emilia‐Romagna accounted for more than 40% of all respondents). Participants reported managing a median of 35 PBC patients annually (range: 10–120), with 75% managing ≥ 20 patients/year. The subsample of 80 clinicians (87% of the initial cohort) that participated in the second round retained similar demographic and professional characteristics to the original group: median age 54 years (range: 28–68), median PBC management experience 21 years (range: 4–42), and comparable regional distribution (Lombardy, Sicily, and Emilia‐Romagna remained overrepresented). Nearly all participants were members of at least one scientific society focused on liver diseases, and 53 (58%) had published papers on PBC within the last decade. In the first round (and with similar distribution in the second) 75 called themselves hepatologists (82%), 7 gastroenterologists (8%), 1 internal medicine specialist (1%) and 9 did not specify their field of activity (10%). Due to the small sample size of the groups other than hepatologists, no statistical tests were applied to assess between‐groups differences. The sample reflects Italian clinical practice, showcasing regional diversity and expertise in PBC management. This highlights the importance of evidence‐based diagnostic protocols and supports the robustness of the consensus findings.

### 3.2 Key Insights From the First Round of the Delphi Questionnaire

Table 1 presents the main topics of the study, and the appropriateness indices evaluated according to the RAND/UCLA Method.

**TABLE 1:** Appropriateness of the statements of Delphi Questionnaire 1 and 2 evaluated according to the RAND/UCLA method.

Domain	Question	Item	Median	Evaluation	Quartile _1	Quartile _3	Inappropriate	Uncertain	Appropriate	IPRCP	AI	IPRAS
Round 1												
Identification and diagnosis	1. ALP alteration in asymptomatic patients without concomitant diseases requires:	IDd01_r1—No further investigation	1	Inappropriate	1	2	84%	4%	12%	1	4	8.35
		IDd01_r2—Medical and pharmacological history	9	Appropriate	7	9	0%	15%	85%	8	3	6.85
		IDd01_r3—Biochemical study of liver function	9	Appropriate	8	9	0%	7%	93%	8.5	3.5	7.6
		IDd01_r4—Virological and autoimmunity screening	9	Appropriate	7	9	3%	13%	84%	8.5	3.5	7.6
		IDd01_r5—Abdominal ultrasound	9	Appropriate	7	9	3%	11%	86%	8.5	3.5	7.6
		IDd01_r6—Biochemical monitoring every 3–6 months	8	Appropriate	7	9	3%	16%	80%	8	3	6.85
	2. The alteration of ALP in a patient with itching and fatigue, without any concomitant conditions, requires:	IDd02_r1—No further investigation	1	Inappropriate	1	1	88%	0%	12%	1	4	8.35
		IDd02_r2—Medical and pharmacological history	9	Appropriate	8	9	0%	7%	93%	8.5	3.5	7.6
		IDd02_r3—Biochemical study of liver function	9	Appropriate	9	9	0%	1%	99%	9	4	8.35
		IDd02_r4—Virological and autoimmunity screening	9	Appropriate	9	9	0%	2%	98%	9	4	8.35
		IDd02_r5—Abdominal ultrasound	9	Appropriate	9	9	0%	4%	96%	9	4	8.35
		IDd02_r6—Biochemical monitoring every 3–6 months	9	Appropriate	8	9	11%	8%	82%	8.5	3.5	7.6
	3. The alteration of ALP in an asymptomatic patient with concomitant pathological conditions (Hashimoto's thyroiditis, Sjögren's syndrome, celiac disease, or systemic sclerosis) requires:	IDd03_r1—No further investigation	1	Inappropriate	1	1	86%	2%	12%	1	4	8.35
		IDd03_r2—Medical and pharmacological history	9	Appropriate	8	9	0%	10%	90%	8.5	3.5	7.6
		IDd03_r3—Biochemical study of liver function	9	Appropriate	9	9	0%	2%	98%	9	4	8.35
		IDd03_r4—Virological and autoimmunity screening	9	Appropriate	9	9	1%	1%	98%	9	4	8.35
		IDd03_r5—Abdominal ultrasound	9	Appropriate	8	9	1%	5%	93%	9	4	8.35
		IDd03_r6—Biochemical monitoring every 3–6 months	9	Appropriate	7	9	9%	8%	84%	8.5	3.5	7.6
	4. The alteration of ALP associated with itching and fatigue in the presence of concomitant pathological conditions requires:	IDd04_r1—No further investigation	1	Inappropriate	1	1	88%	0%	12%	1	4	8.35
		IDd04_r2—Medical and pharmacological history	9	Appropriate	8	9	1%	4%	95%	8.5	3.5	7.6
		IDd04_r3—Biochemical study of liver function	9	Appropriate	9	9	0%	0%	100%	9	4	8.35
		IDd04_r4—Virological and autoimmunity screening	9	Appropriate	9	9	0%	1%	99%	9	4	8.35
		IDd04_r5—Abdominal ultrasound	9	Appropriate	9	9	0%	3%	97%	9	4	8.35
		IDd04_r6—Biochemical monitoring every 3–6 months	9	Appropriate	8	9	11%	5%	84%	9	4	8.35
	5. The evidence of chronic cholestasis (elevated ALP and GGT and/or bilirubin > 6 months), regardless of symptoms, initially requires:	IDd05_r1—Pathological history, remote and pharmacological	9	Appropriate	8	9	2%	5%	92%	8.5	3.5	7.6
		IDd05_r2—Virological screening and autoimmunity	9	Appropriate	9	9	0%	5%	95%	9	4	8.35
		IDd05_r3—Ultrasound of the abdomen	9	Appropriate	9	9	0%	1%	99%	9	4	8.35
		IDd05_r4—Measurement of liver stiffness by VCTE	9	Appropriate	6.75	9	3%	22%	75%	8	3	6.85
	6. The evidence of chronic cholestasis (elevated ALP and GGT and/or bilirubin > 6 months), regardless of symptoms, when first‐level tests previously listed are inconclusive, requires:	IDd06_r1—MRCP ± EUS	8	Appropriate	7	9	9%	12%	79%	8	3	6.85
		IDd06_r2—Liver biopsy	8	Appropriate	7	9	5%	17%	77%	8	3	6.85
		IDd06_r3—Genetic study (i.e., ATP8B1, ABCB11, ABCB4)	5	Uncertain	3	7	30%	38%	32%	5.15	0.15	2575
	7. The detection of AMA+ in the absence of chronic cholestasis (elevated ALP and GGT and/or bilirubin > 6 months), regardless of symptoms, initially requires:	IDd07_r1—Search for other concomitant autoimmune diseases	8	Appropriate	6	9	9%	18%	73%	8	3	6.85
		IDd07_r2—Ultrasound of the abdomen	8	Appropriate	5	9	16%	20%	64%	7.5	2.5	6.1
		IDd07_r3—Measurement of liver stiffness by VCTE	6	Uncertain	4.75	8	23%	28%	49%	6.5	1.5	4.6
		IDd07_r4—Annual biohumoral monitoring	9	Appropriate	8	9	9%	4%	87%	9	4	8.35
	8. The detection of AMA+ in the absence of chronic cholestasis (elevated ALP and GGT and/or bilirubin > 6 months), regardless of symptoms, when the previously listed first‐level tests are inconclusive, requires	IDd08_r1—MRCP ± EUS	3	Inappropriate	1	5.25	59%	17%	24%	3	2	5.35
		IDd08_r2—Liver biopsy	3	Inappropriate	1	7	51%	22%	27%	3	2	5.35
		IDd08_r3—Genetic study (i.e., ATP8B1, ABCB11, ABCB4)	3	Inappropriate	1	5	57%	30%	13%	3	2	5.35
	9. The detection of AMA+ in the presence of chronic cholestasis (elevated ALP and GGT and/or bilirubin > 6 months), regardless of symptoms, initially requires	IDd09_r1—Search for other concomitant autoimmune diseases	9	Appropriate	7	9	2%	11%	87%	8.5	3.5	7.6
		IDd09_r2—Ultrasound of the abdomen	9	Appropriate	8	9	0%	4%	96%	9	4	8.35
		IDd09_r3—Measurement of liver stiffness by VCTE	9	Appropriate	8	9	3%	9%	88%	8.5	3.5	7.6
Identification and diagnosis	10. The detection of AMA+ in the presence of chronic cholestasis (elevated ALP and GGT and/or bilirubin > 6 months), regardless of symptoms, when the previously listed first‐level tests are inconclusive, requires:	IDd10_r1—MRCP ± EUS	7	Appropriate	4	9	25%	24%	52%	6.5	1.5	4.6
		IDd10_r2—Liver biopsy	7	Appropriate	4	9	24%	19%	57%	7	2	5.35
		IDd10_r3—Genetic study (i.e., ATP8B1, ABCB11, ABCB4)	4	Uncertain	1	6	44%	31%	25%	4	1	3.85
	11. The detection of ANA+ (anti‐sp100, anti‐gp210, anti‐centromere) in the absence of chronic cholestasis (elevated ALP and GGT and/or bilirubin > 6 months), regardless of symptoms, initially requires:	IDd11_r1—Screening for other concomitant autoimmune diseases	8	Appropriate	7	9	7%	12%	81%	8	3	6.85
		IDd11_r2—Abdominal ultrasound	7	Appropriate	5	9	11%	24%	65%	7.5	2.5	6.1
		IDd11_r3—Measurement of liver stiffness via VCTE	7	Appropriate	5	8	22%	26%	52%	6.5	1.5	4.6
		IDd11_r4—Annual biomarker monitoring	9	Appropriate	8	9	2%	10%	88%	8.7	3.7	7.9
	12. The detection of ANA+ (anti‐sp100, anti‐gp210, anti‐centromere) in the absence of chronic cholestasis (elevated ALP and GGT and/or bilirubin > 6 months), regardless of symptoms, when the previously listed first‐level tests are inconclusive, requires:	IDd12_r1—MRCP ± EUS	3	Inappropriate	1	6	56%	20%	24%	3	2	5.35
		IDd12_r2—Liver biopsy	3	Uncertain	1	6	52%	24%	25%	3.5	1.5	4.6
		IDd12_r3—Genetic testing (i.e., ATP8B1, ABCB11, ABCB4)	2	Inappropriate	1	5	62%	24%	15%	2.5	2.5	6.1
	13. The detection of ANA+ (anti‐sp100, anti‐gp210, anti‐centromere) in the presence of chronic cholestasis (elevated ALP and GGT and/or bilirubin > 6 months), regardless of symptoms, requires:	IDd13_r1—Screening for other concomitant autoimmune diseases	9	Appropriate	7	9	3%	13%	83%	8.5	3.5	7.6
		IDd13_r2—Abdominal ultrasound	9	Appropriate	9	9	1%	2%	97%	9	4	8.35
		IDd13_r3—Measurement of liver stiffness via VCTE	9	Appropriate	8	9	6%	9%	85%	8.5	3.5	7.6
		IDd13_r4—MRCP ± EUS	5	Uncertain	2	8	34%	33%	34%	5	0	2.35
		IDd13_r5—Liver biopsy	5	Uncertain	3	8	37%	26%	37%	5.3	0.3	2.8
	14. The detection of ANA+ (anti‐sp100, anti‐gp210, anti‐centromere) in the presence of chronic cholestasis, irrespective of symptomatology, where the first‐level investigations listed above have not been conclusive, requires:	IDd14_r1—MRCP ± EUS	6	Uncertain	3	9	27%	26%	47%	6	1	3.85
		IDd14_r2—Liver biopsy	8	Appropriate	5	9	17%	15%	69%	7.5	2.5	6.1
		IDd14_r3—Genetic testing (i.e., ATP8B1, ABCB11, ABCB4)	5	Uncertain	1	6	38%	38%	24%	4	1	3.85
	15. The detection of chronic cholestasis (elevated ALP and GGT and/or bilirubin > 6 months) in the presence of AMA/ANA, regardless of symptoms, requires:	IDd15_r1—Biomarker monitoring every 3–6 months	9	Appropriate	8	9	8%	10%	82%	8.5	3.5	7.6
		IDd15_r2—Abdominal ultrasound	9	Appropriate	9	9	0%	3%	97%	9	4	8.35
		IDd15_r3—Measurement of liver stiffness via VCTE	9	Appropriate	8	9	2%	10%	88%	8.5	3.5	7.6
		IDd15_r4—MRCP ± EUS	6	Uncertain	3	7	29%	36%	35%	5.5	0.5	3.1
		IDd15_r5—Liver biopsy	5	Uncertain	3	7	29%	39%	31%	5.5	0.5	3.1
		IDd15_r6—Re‐testing for autoimmunity every 6–12 months	5	Uncertain	2	8	38%	24%	38%	5.5	0.5	3.1
		IDd15_r7—Genetic testing (i.e., ATP8B1, ABCB11, ABCB4)	3	Inappropriate	1	5	55%	35%	10%	3	2	5.35
	16. In the presence of a clinical diagnosis of PBC (chronic cholestasis and AMA+), staging of fibrosis and the potential presence of portal hypertension requires:	IDd16_r1—Albumin and platelet dosage	9	Appropriate	9	9	1%	6%	93%	9	4	8.35
		IDd16_r2—EGDS	7	Appropriate	5	9	13%	29%	57%	7	2	5.35
		IDd16_r3—Abdominal ultrasound	9	Appropriate	9	9	0%	2%	98%	9	4	8.35
		IDd16_r4—Measurement of liver stiffness via VCTE	9	Appropriate	9	9	1%	4%	94%	9	4	8.35
		IDd16_r5—Liver biopsy	4	Uncertain	2	5	47%	38%	15%	3.5	1.5	4.6
		IDd16_r6—Not necessary	1	Inappropriate	1	1	93%	1%	6%	1	4	8.35
	17. In which of the following settings is a liver biopsy required:	IDd17_r1—Disease staging and degree of fibrosis	5	Uncertain	3	8	27%	34%	39%	5.5	0.5	3.1
		IDd17_r2—Cholestasis without autoantibodies	8	Appropriate	7	9	6%	19%	75%	8	3	6.85
		IDd17_r3—Suspected ductopenic variant	9	Appropriate	7	9	4%	17%	79%	8	3	6.85
		IDd17_r4—Overlap syndrome	9	Appropriate	9	9	2%	2%	96%	9	4	8.35
		IDd17_r5—Coexistence of other etiologies (i.e., MASLD)	8	Appropriate	7	9	6%	13%	81%	8.5	3.5	7.6
		IDd17_r6—Poor response to UDCA	6	Uncertain	4	8	21%	37%	42%	5.7	0.7	3.4
	18. VCTE can be used for:	IDd18_r1—Staging of the disease and degree of fibrosis along with biopsy	6	Uncertain	4	9	24%	27%	49%	6.5	1.5	4.6
		IDd18_r2—Prognosis assessment	8	Appropriate	6	9	12%	16%	72%	8	3	6.85
		IDd18_r3—Staging of the disease and degree of fibrosis instead of biopsy	8	Appropriate	7	9	6%	16%	79%	8	3	6.85
		IDd18_r4—Only for the diagnosis of advanced fibrosis and cirrhosis	4	Uncertain	2	6	46%	30%	24%	4	1	3.85
		IDd18_r5—Non‐invasive monitoring of fibrosis	9	Appropriate	8	9	0%	8%	92%	8.5	3.5	7.6
Treatment and follow‐up	1. UDCA in PBC is used:	TFd01_r1—During pregnancy and breastfeeding	9	Appropriate	7	9	10%	9%	81%	8	3	6.85
		TFd01_r2—In a single dose	1	Inappropriate	1	3	77%	13%	10%	1.95	3.05	6925
		TFd01_r3—In fractionated doses	9	Appropriate	9	9	1%	1%	98%	9	4	8.35
		TFd01_r4—After main meals	9	Appropriate	7	9	7%	9%	84%	8.5	3.5	7.6
	2. The assessment of response to therapy is performed:	TFd02_r1—6 months after starting UDCA	8	Appropriate	6	9	7%	24%	69%	7.55	2.55	6175
		TFd02_r2—12 months after starting UDCA	9	Appropriate	5	9	19%	15%	66%	7.5	2.5	6.1
		TFd02_r3—Through Measurement of ALP, GGT, Bilirubin, and Transaminases	9	Appropriate	9	9	0%	0%	100%	9	4	8.35
	3. Referral to a centre with specific clinical expertise and research on PBC is required if:	TFd03_r1—Overlap syndrome	8	Appropriate	7	9	10%	14%	76%	8	3	6.85
		TFd03_r2—No Response/partial response to UDCA	8	Appropriate	7	9	10%	13%	77%	8	3	6.85
		TFd03_r3—Coexistence of other etiologies (i.e., MASLD)	7	Appropriate	5	9	15%	28%	57%	7	2	5.35
		TFd03_r4—Coexistence of other associated autoimmune diseases	7	Appropriate	5	9	14%	26%	60%	7.5	2.5	6.1
	4. In the case of absent or partial response to UDCA, consideration should be given to:	TFd04_r1—Overlap syndrome	8	Appropriate	6,75	9	6%	19%	75%	8	3	6.85
		TFd04_r2—Coexistence of other etiologies (i.e., MASLD)	7	Appropriate	6	9	3%	23%	74%	7.5	2.5	6.1
		TFd04_r3—ductopenic variant	8	Appropriate	6,75	9	5%	20%	75%	7.5	2.5	6.1
		TFd04_r4—Potential ductal reaction	7	Appropriate	5	8	13%	35%	52%	6.5	1.5	4.6
		TFd04_r5—Diagnostic workup (i.e., MRCP ± EUS, liver biopsy) at their own centre	8	Appropriate	6	9	8%	23%	69%	7.05	2.05	5425
		TFd04_r6—Referral to a centre with specific clinical and research expertise in PBC	8	Appropriate	5,75	9	14%	18%	68%	7.5	2.5	6.1
	5. At the time of PBC diagnosis and during follow‐up, it is recommended:	TFd05_r1—Annual measurement of vitamin D, calcium, and PTH	9	Appropriate	8	9	0%	5%	95%	9	4	8.35
		TFd05_r2—DEXA every 1–4 years	9	Appropriate	8	9	0%	3%	97%	9	4	8.35
		TFd05_r3—Screening for Sjögren's syndrome, systemic sclerosis, and celiac disease	9	Appropriate	7	9	0%	13%	88%	8.5	3.5	7.6
		TFd05_r4—Measurement of TSH	9	Appropriate	8	9	2%	9%	89%	8.5	3.5	7.6
		TFd05_r5—Measurement of total cholesterol	9	Appropriate	8	9	0%	1%	99%	9	4	8.35
		TFd05_r6—Assessment of cardiovascular risk	8	Appropriate	7	9	0%	20%	80%	8	3	6.85
		TFd05_r7—Referral to the specialist	8	Appropriate	7	9	6%	17%	77%	8.5	3.5	7.6
	6. Follow‐up in the primary care setting is expected:	TFd06_r1—Non‐cirrhotic patients	7	Appropriate	5	8	19%	27%	53%	6.5	1.5	4.6
		TFd06_r2—Asymptomatic patients	6	Uncertain	4	8	20%	34%	45%	6.5	1.5	4.6
		TFd06_r3—Patients responsive to treatment with UDCA	7	Appropriate	5	8	19%	24%	57%	6.55	1.55	4675
		TFd06_r4—In all cases of PBC	2	Uncertain	1	8	60%	9%	31%	3.9	1.1	4
		TFd06_r5—Normal liver tests for at least 12 months	5	Uncertain	4	8	23%	40%	38%	5.55	0.55	3175
	7. The performance of EGDS for screening of portal hypertension in patients with PBC is recommended if:	TFd07_r1—Histologically documented cirrhosis	8	Appropriate	5	9	14%	27%	59%	7.5	2.5	6.1
		TFd07_r2—If LSM ≥ 20 kPa or PLT < 150 000/mm^3^	9	Appropriate	8,75	9	1%	9%	90%	9	4	8.35
		TFd07_r3—If LSM ≥ 20 kPa or PLT < 150 000/mm^3^ in the absence of NSBB	9	Appropriate	6	9	15%	11%	74%	8	3	6.85
		TFd07_r4—In Follow‐up according to the Baveno VI criteria	9	Appropriate	8	9	6%	7%	88%	8.55	3.55	7675
		TFd07_r5—If Ultrasound signs of portal hypertension are present	9	Appropriate	7	9	6%	9%	85%	8.5	3.5	7.6
	8. The role of obeticholic acid in PBC:	TFd08_r1—Only as second line after UDCA	9	Appropriate	8	9	5%	6%	90%	8.5	3.5	7.6
		TFd08_r2—In Compensated cirrhosis (Child‐Pugh A)	9	Appropriate	7	9	8%	10%	82%	8.4	3.4	7.45
		TFd08_r3—Initial dosage of 5 mg up to 10 mg	9	Appropriate	9	9	0%	0%	100%	9	4	8.35
		TFd08_r4—Requires discontinuation if side effects appear	7	Appropriate	5	9	14%	26%	60%	7	2	5.35
		TFd08_r5—May be useful to introduce before 12 months after starting UDCA if intolerance/insufficient response to UDCA	8	Appropriate	7	9	3%	17%	79%	8	3	6.85
	9. Given the current state of knowledge, regardless of approved indications, the use of bezafibrate in PBC can be considered:	TFd09_r1—Always in conjunction with UDCA	8	Appropriate	7	9	9%	13%	78%	8	3	6.85
		TFd09_r2—For the treatment of compensated cirrhosis	7	Appropriate	5	8	18%	29%	53%	6.5	1.5	4.6
		TFd09_r3—For the reduction of mortality in PBC	6	Uncertain	5	8	20%	31%	49%	6	1	3.85
		TFd09_r4—Requires monitoring of creatinine	7	Appropriate	5	9	13%	25%	62%	7	2	5.35
		TFd09_r5—Use in other contexts (i.e., in association with OCA)	7	Appropriate	5	9	11%	25%	63%	7	2	5.35
Treatment and follow‐up	10. Given the current state of knowledge, regardless of approved indications, the use of budesonide in PBC can be considered:	TFd10_r1—Always in combination with UDCA	7	Uncertain	3,5	9	25%	23%	52%	6.5	1.5	4.6
		TFd10_r2—Only in non‐cirrhotic patients	7	Uncertain	4	9	22%	21%	57%	6.5	1.5	4.6
		TFd10_r3—In the case of active hepatic inflammation	7	Appropriate	5	8	17%	26%	56%	6.5	1.5	4.6
		TFd10_r4—In the presence of autoimmune hepatitis	8	Appropriate	6	9	8%	21%	71%	8	3	6.85
		TFd10_r5—Only for non‐long‐term treatments	5	Uncertain	3	7	32%	38%	30%	4.6	0.4	2.95
	11. In the case of PBC in pregnant women:	TFd11_r1—The use of UDCA is recommended throughout pregnancy	9	Appropriate	8	9	6%	7%	87%	8.5	3.5	7.6
		TFd11_r2—Cholestyramine and rifampicin are indicated in the third trimester	6	Uncertain	2	8	32%	20%	48%	5.5	0.5	3.1
		TFd11_r3—The use of plasma exchange is indicated if the itching is untreatable	7	Appropriate	5	8	15%	34%	51%	6.5	1.5	4.6
		TFd11_r4—Supplementation of fat‐soluble vitamins is mandatory	8	Appropriate	6	9	5%	24%	71%	8	3	6.85
		TFd11_r5—Referral to a centre with specific clinical and research expertise in PBC is necessary	9	Appropriate	7,5	9	8%	8%	84%	8.5	3.5	7.6
	12. The management of mild pruritus in PBC includes:	Tfd12_R1—The use of cholestyramine as first‐line	9	Appropriate	7	9	3%	9%	87%	8.5	3.5	7.6
		TFd12_r2—The use of bezafibrate and rifampicin only if intolerance or no response to cholestyramine	8	Appropriate	6,5	9	7%	18%	75%	8	3	6.85
		TFd12_r3—The management by the primary care physician	3	Inappropriate	1	5	61%	28%	11%	3	2	5.35
	13. The management of moderate–severe pruritus in PBC includes:	TFd13_r1—The use of cholestyramine as first‐line	9	Appropriate	8	9	1%	9%	90%	8.5	3.5	7.6
		TFd13_r2—The use of bezafibrate in all cases except decompensated cirrhosis.	7	Appropriate	5	9	9%	30%	61%	7	2	5.35
		TFd13_r3—The use of rifampicin in cases of intolerance or non‐response to bezafibrate	7	Appropriate	6	9	5%	22%	74%	7.5	2.5	6.1
		TFd13_r4—The use of naltrexone or sertraline in cases of intolerance or contraindications to both bezafibrate and rifampicin	8	Appropriate	7	9	0%	18%	82%	7.6	2.6	6.25
		TFd13_r5—Referral to a centre with specific clinical and research experience in PBC and OLT evaluation	9	Appropriate	8	9	3%	7%	90%	8.5	3.5	7.6
	14. Referral to a center with specific clinical expertise and research on PBC for potential liver transplantation evaluation is indicated if:	TFd14_r1—In all cases of MELD > 15	9	Appropriate	8	9	1%	3%	95%	8.5	3.5	7.6
		TFd14_r2—Presence of complications of liver cirrhosis, including HCC	9	Appropriate	9	9	1%	2%	97%	9	4	8.35
		TFd14_r3—Persistently elevated total bilirubin > 3 mg/dL	8	Appropriate	7	9	9%	15%	76%	8	3	6.85
		TFd14_r4—Severe pruritus resistant to medical therapy regardless of MELD	9	Appropriate	8	9	3%	3%	93%	9	4	8.35
Round 2												
Identification and diagnosis	1. Genetic testing (i.e., ATP8B1, ABCB11, ABCB4) is required:	IDd1_r1—In the case of diagnostic uncertainty from liver biopsy	6	Uncertain	4	8	19%	34%	48%	6.15	1.15	4075
		IDd1_r2—In the case of diagnostic uncertainty on MRCP ± EUS	5	Uncertain	2	7	38%	30%	33%	5	0	2.35
		IDd1_r3—In both of the above cases	5,5	Uncertain	3	7,25	36%	24%	40%	5	0	2.35
		IDd1_r4—Regardless of the results of the previous tests in cases of strong clinical suspicion	6	Uncertain	4	8	24%	36%	40%	5.65	0.65	3325
		IDd1_r5—In all cases of chronic cholestasis of NDD regardless of the positivity of AMA/ANA	3	Inappropriate	2	6	54%	23%	24%	3.5	1.5	4.6
	2. A liver biopsy is indicated:	IDd2_r1—For AMA+ or ANA+ in the absence of cholestasis	2	Inappropriate	1	5	59%	21%	20%	3	2	5.35
		IDd2_r2—For AMA+ or ANA+ in the presence of chronic cholestasis as a first‐line approach	2	Inappropriate	1	5	68%	18%	15%	2.65	2.35	5875
		IDd2_r3—For AMA+ or ANA+ in the presence of chronic cholestasis in case of doubt after other diagnostic investigations (abdominal ultrasound, VCTE, MRCP ± EUS)	7	Appropriate	3	8	28%	19%	54%	6.5	1.5	4.6
		IDd2_r4—Always for fibrosis staging	3	Inappropriate	1	5	63%	26%	11%	3	2	5.35
	3. VCTE can be used to:	IDd3_r1—For disease staging and degree of fibrosis along with biopsy	6	Uncertain	3	8	29%	33%	39%	5.5	0.5	3.1
		IDd3_r2—For disease staging and degree of fibrosis regardless of biopsy	8	Appropriate	7	9	8%	16%	76%	8	3	6.85
		IDd3_r3—Only for the identification and monitoring of advanced fibrosis	5	Uncertain	3	7	36%	33%	31%	5	0	2.35
		Dd3_r4—For fibrosis staging if AMA+ or ANA+ in the absence of chronic cholestasis	5	Uncertain	2	7	34%	29%	38%	5	0	2.35
		IDd3_r5—For fibrosis staging if AMA+ or ANA+ in the presence of chronic cholestasis	8	Appropriate	5	9	9%	21%	70%	7.35	2.35	5875
Treatment and follow‐up	1. The management by the General Practitioner in PBC is expected for:	TFd1_r1—Non‐cirrhotic patients.	4	Uncertain	2	6	49%	33%	18%	4	1	3.85
		TFd1_r2—Asymptomatic patients	4	Uncertain	2	6	48%	33%	19%	4	1	3.85
		TFd1_r3—Patients responsive to treatment with UDCA	5	Uncertain	3	6,5	38%	37%	25%	4.5	0.5	3.1
		TFd1_r4—In all cases of PBC	1	Inappropriate	1	3	87%	10%	3%	1.5	3.5	7.6
		TFd1_r5—Normal liver tests for at least 12 months	5	Uncertain	2	6	41%	37%	23%	4.5	0.5	3.1
		TFd1_r6—Not required	5	Uncertain	2	8	42%	19%	39%	4.8	0.2	2.65
	2. Continuous evaluation and regular follow‐up of the patient should be performed:	TFd2_r1—At least every 3 months in patients with a high risk of disease progression	6	Uncertain	3,5	8	25%	36%	38%	5.6	0.6	3.25
		TFd2_r2—At least every 6 months in patients with intermediate/high risk of disease progression	8	Appropriate	8	9	2%	13%	85%	8.5	3.5	7.6
		TFd2_r3—At least once a year in patients with low risk of disease progression	8	Appropriate	7	9	2%	11%	87%	8.5	3.5	7.6
		TFd2_r4–6 months after the start of UDCA therapy to schedule the potential second‐line initiation visit at 12 months	8	Appropriate	7	9	2%	9%	89%	8.5	3.5	7.6

Regarding the identification, diagnosis, and staging of PBC patients, consensus was reached on nearly all items, with medians ≥ 7 or ≤ 3 and limited variability among raters. In most cases, the assessments or diagnostic procedures described by the items were deemed appropriate. For instance, in the case of chronic cholestasis (elevation of ALP and GGT and/or bilirubin lasting more than 6 months), regardless of symptoms, the following first‐line procedures were considered consistently highly appropriate: Liver stiffness measurement by VCTE, Abdominal ultrasound, Virological and autoimmunity screening. Whenever such procedures were not decisive, liver biopsy and MRCP ± EUS were judged consistently appropriate. This clear delineation of appropriate diagnostic strategies emphasises the value of precise, evidence‐based protocols. Building on this diagnostic framework, the study explored consensus on treatment strategies, focusing on first‐ and second‐line therapies for PBC.

With regard to the effects of treatments, changes in ALP, GGT, bilirubin, and transaminase levels were deemed highly appropriate (only two raters gave score 7 and 8, whilst all others the maximum score 9).

A consistent finding was that “no in‐depth diagnostics” was considered inappropriate in all cases with ALP alterations.

In a clinical scenario characterised by “AMA+ in the absence of chronic cholestasis (elevation of ALP and GGT and/or bilirubin > 6 months), regardless of the symptoms, where first level tests have not been conclusive”, the procedures “MRCP ± EUS”, “Liver biopsy”, and “Genetic study (i.e., ATP8B1, ABCB11, ABCB4)” were judged to be inappropriate.

Similarly, when “ANA+ (anti‐sp100, anti‐gp210, anti‐centromere) is detected in the absence of chronic cholestasis (elevation of ALP and GGT and/or bilirubin > 6 months), regardless of symptoms, and first‐level tests have not been conclusive”, the same procedures – “MRCP ± EUS” and “Genetic study (i.e., ATP8B1, ABCB11, ABCB4)” – were also deemed inappropriate.

Genetic studies were regarded as inappropriate even in case of chronic cholestasis (elevation of ALP and GGT and/or bilirubin > 6 months) with the presence of AMA/ANA, regardless of symptoms. Additionally, the “do not perform the staging of fibrosis and the possible presence of portal hypertension in presence of a clinical diagnosis of PBC” was considered inappropriate.

Regarding treatment appropriateness, the consensus confirmed UDCA as the recommended first‐line therapy for PBC, with administration in divided doses and taken with meals receiving a high consensus (median = 9, IQR = 7–9). To assess the response to UDCA, a biochemical evaluation at 6 and 12 months was considered appropriate (median = 8 for 6 months, IQR = 6–9; median = 9 for 12 months, IQR = 5–9), prioritising the measurement of ALP, GGT, bilirubin, and transaminases (median = 9, IQR = 9–9) as key indicators of treatment efficacy. Biochemical monitoring at 6 and 12 months was strongly endorsed to assess treatment efficacy and guide decisions on initiating second‐line therapies such as OCA. For second‐line therapy, OCA was recommended exclusively for patients with an inadequate UDCA response or those intolerant to UDCA, with consensus for use in a compensatory cirrhosis setting (Child‐Pugh A) and initial dosing of 5–10 mg (median = 9, IQR = 8–9).

OCA may be considered earlier than the standard 12‐month evaluation if UDCA intolerance or inadequate response is observed (median = 8, IQR = 7–9). This staged therapeutic approach aligns with expert guidelines, aiming to optimise efficacy while managing tolerability concerns. While treatment consensus was strong, variability emerged around genetic studies and invasive procedures like liver biopsy, prompting further exploration in the second Delphi round.

Consensus on pruritus management strategies varied according to severity. For mild pruritus, high consensus was reached on using cholestyramine as the first‐line treatment (median = 9, IQR = 7–9) and limiting bezafibrate and rifampicin use to cases of intolerance or non‐response to cholestyramine (median = 8, IQR = 7–9). For moderate‐to‐severe pruritus, the panel strongly recommended cholestyramine as first‐line therapy (median = 9, IQR = 8–9) and, when necessary, referral to specialised centres for liver transplant evaluation (median = 9, IQR = 8–9). A stepwise approach was endorsed for cases unresponsive to cholestyramine: rifampicin as second‐line therapy in bezafibrate‐intolerant patients (median = 7, IQR = 6–9), and naltrexone or sertraline if both bezafibrate and rifampicin were contraindicated or ineffective (median = 8, IQR = 7–9). Additionally, bezafibrate was deemed inappropriate for use in decompensated cirrhosis (median = 7, IQR = 5–9).

Pruritus management in special populations also deserved particular attention. In pregnant women with intractable pruritus, plasma exchange was considered appropriate (median = 7, IQR = 5–8).

### 3.3 Key Insights From the Second Round of the Delphi Questionnaire

For 17 items (spanning 10 topics), clinicians' responses resulted in uncertainty. This occurred when the medians fell within the 4–6 range, but in some cases, even medians below or above 6 were classified as “uncertain” due to substantial variability among raters.

In accordance with the Delphi method, the findings from the first round were presented to all responders, and some items for which consensus was not reached were resubmitted to the same sample. A total of 80 clinicians (87% of the initial 92) responded to the second round. The subsample that participated in the second round was closely representative of the original group in terms of age and experience in managing PBC.

As several items related to genetic studies resulted in “uncertainty”, the scientific board of the study decided to rephrase these items. Instead of assessing the relevance of the utility of genetic studies (among other assessments) in specific clinical scenarios, the second questionnaire (Q2) posed the question in terms of which clinical situations should require genetic studies (ATP8B1, ABCB11, ABCB4). However, even with this revision, clinicians provided divergent answers, and uncertainty persisted.

The same approach was applied to liver biopsy. In the second round, this procedure was considered inappropriate for patients with AMA+ or ANA+ in the absence of cholestasis, or in the presence of chronic cholestasis as an initial assessment. However, uncertainty persisted for patients with AMA+ or ANA+ in the presence of chronic cholestasis when there was doubt regarding other diagnostic tests (abdominal ultrasound, VCTE, MRCP ± EUS) or when staging fibrosis was required. Genetic studies and liver biopsy were sources of notable variability, particularly in cases with AMA+ or ANA+ without chronic cholestasis. Similarly, questions around the combined use of VCTE and biopsy for fibrosis monitoring yielded inconclusive results, highlighting the need for additional research to refine these recommendations.

The timing of follow‐up visits and their alignment with patient risk profiles was examined next, with a focus on balancing surveillance intervals with disease progression risks.

VCTE was considered appropriate for staging disease and assessing fibrosis degree, regardless of biopsy, and for staging fibrosis in patients with AMA+ or ANA+ in the presence of chronic cholestasis. Uncertainty remained regarding the following items:

- The use of VCTE for staging disease and fibrosis in conjunction with biopsy;
- Identification and monitoring advanced fibrosis;
- Staging fibrosis in cases where AMA+ or ANA+ was present in the absence of chronic cholestasis.

Management of PBC by the General Practitioner is considered inappropriate in all cases of PBC.

Regarding the timing of follow‐up visits, there was uncertainty about scheduling visits at least every 3 months for patients at high risk of disease progression. On the other hand, clinicians judged it appropriate to perform visits:

- At least every 6 months in case of patients with intermediate/high risk of disease progression;
- At least once a year in patients with low risk of disease progression;
- After 6 months from the start of UDCA therapy to schedule a visit for potential initiation of second‐line therapy at 12 months.

Consensus was achieved for follow‐up visits stratified by patient risk level, with intervals ranging from 6 months for higher‐risk cases to annual visits for low‐risk patients. However, uncertainty regarding the appropriateness of visits every 3 months for high‐risk patients underscores the challenge of standardising follow‐up care across varying clinical contexts.

## 4 Discussion

This Delphi Consensus study focuses on developing a set of quality measures specifically aimed at improving the diagnosis, management, and treatment of PBC. The following analysis highlights the areas of agreement among respondents, primarily concerning the identification and diagnostic evaluation of PBC patients.

The consensus strongly supports specific diagnostic tools and monitoring protocols for patients with biochemical abnormalities like elevated ALP or GGT. VCTE, abdominal ultrasound, and autoimmune and virological screening were identified as highly appropriate first‐line tools for diagnosing chronic cholestasis, while genetic studies and MRCP ± EUS were deemed inappropriate for AMA+ or ANA+ cases without cholestasis. For asymptomatic patients, biochemical monitoring, liver function tests, and ultrasound were considered appropriate (median score: 9) [13, 14]. Comprehensive protocols, including ultrasound, virological screening, and autoimmune assessment, were emphasised for patients with pruritus or fatigue. Accurate measurement of ALP and bilirubin is critical for evaluating treatment response (UDCA and UDCA+OCA), ensuring timely and effective clinical decisions [15]. The subsequent analysis addresses areas of uncertainty, focusing primarily on diagnostic challenges where consensus was not achieved.

### 4.1 Non‐Invasive Vs. Invasive Diagnostic Tools

For patients with chronic cholestasis (characterised by persistent ALP and GGT elevations lasting over 6 months), the Delphi consensus strongly endorsed VCTE as an essential non‐invasive tool for fibrosis staging. VCTE was widely recognised as a valuable alternative to liver biopsy, particularly given its safety, accessibility, and ability to reduce the need for invasive procedures in routine practice.

However, despite its advantages, several areas of uncertainty remain. These include its diagnostic utility when used in conjunction with liver biopsy, its accuracy in monitoring advanced fibrosis progression, and its role in staging fibrosis in patients with AMA+ or ANA+ serology in the absence of biochemical cholestasis. These limitations underscore the need for further validation of VCTE across specific subgroups and clinical contexts [13, 14].

The role of liver biopsy, while diminished in routine assessment, remains indispensable in selected cases, particularly when non‐invasive findings are inconclusive or clinical suspicion is high. Biopsy continues to provide critical histological information for diagnosis confirmation, disease staging, and treatment planning, especially in patients with atypical presentations, overlap syndromes, or suspected rapidly progressive liver damage. These scenarios highlight the complementary nature of invasive and non‐invasive diagnostics and the importance of individualised clinical decision‐making based on patient characteristics and resource availability.

Ultimately, while VCTE is positioned as a frontline tool in fibrosis assessment, its integration with—or substitution by—biopsy should be tailored to specific diagnostic challenges. The consensus calls for further research to refine the respective roles of these modalities and support the development of evidence‐based algorithms that balance diagnostic accuracy, patient safety, and healthcare resource optimisation.

However, potential barriers to the adoption of these measures include the need for specialised training to ensure accurate use of VCTE and the limited access to second‐line therapies, such as OCA, in certain geographic regions, particularly in resource‐constrained settings.

Regarding genetic testing, the uncertainty stems from limited evidence supporting its clinical utility, the relatively high cost of these analyses, and the low prevalence of genetic markers directly applicable to PBC management [16]. However, specific clinical scenarios, such as patients with atypical presentations, overlap syndromes, or suspected genetic cholestasis, may benefit from targeted genetic testing. Future research should focus on identifying these patient subgroups to ensure the judicious application of genetic testing in clinical practice [3].

Treatment strategies for PBC remain a widely debated topic. The consensus strongly supports UDCA as the first‐line therapy for PBC, with biochemical response evaluations at 6 and 12 months focusing on ALP, GGT, bilirubin, and transaminase levels [13, 14]. Divided dosing and meal‐time administration were recommended to enhance absorption and adherence [17]. For inadequate UDCA responses or UDCA intolerant patients, OCA was endorsed as second‐line therapy, including compensated cirrhosis (Child‐Pugh A). Follow‐up intervals were stratified by patient risk level, with annual visits ranging from 6 months for intermediate/high‐risk cases to annual visits for low‐risk patients. However, uncertainty arose regarding three‐monthly follow‐ups for high‐risk cases. Regular monitoring enables early progression to second‐line therapy [16], with flexibility to initiate treatment timely in cases of intolerance or partial response.

Recent evidence suggests that OCA improves transplant‐free survival and reduces hepatic decompensation risk (hazard ratio 0.42), particularly in patients at risk of severe liver events [16, 18, 19, 20]. Recent reimbursement restrictions for OCA in some regions may limit its accessibility, necessitating pragmatic alternatives. Clinicians may prioritise bezafibrate (where available) or participate in clinical trials for investigational PPAR agonists. Advocacy for policy revisions, supported by real‐world evidence of OCA's cost‐effectiveness in reducing liver‐related events, is critical to ensure equitable patient access.

Real‐world data validate trial findings, with 42.9% achieving POISE criteria responses at 12 months but only 11% attaining full biochemical normalisation, underscoring the need for additional therapeutic strategies [17]. While the POISE criteria are widely referenced, their validation as a universal response metric remains limited. Further studies are needed to confirm their applicability across diverse populations and treatment regimens. In patients with an inadequate response to UDCA alone, adding bezafibrate off‐label has shown a higher rate of complete biochemical response compared to UDCA monotherapy [21]. As a result, bezafibrate is often prescribed off‐label as a second‐line treatment. Combining OCA and fibrates, such as bezafibrate, demonstrated enhanced efficacy, including significant ALP reduction and higher biochemical normalisation rates in advanced disease stages [22]. Biomarkers like ALP, bilirubin, and ALT remain critical for treatment monitoring [16, 17, 22]. The consensus did not resolve uncertainties around ALP cutoffs for defining UDCA failure, optimal timing for second‐line therapies, or treatment targets (e.g., ALP normalisation vs. < 1.5 × ULN). These gaps reflect evolving evidence and highlight the need for prospective studies to validate criteria such as the POISE trial score. Additionally, emerging therapies like PPAR agonists (e.g., seladelpar, elafibranor) were not included due to their recent approval post‐consensus. Future updates should integrate these agents and address sequencing strategies for triple therapy (UDCA + OCA + PPAR agonists).

Fourth, the consensus highlighted pruritus as a critical management issue in PBC, significantly impacting quality of life and being a primary cause of patient distress [17, 23]. Real‐world data from Italian and Japanese cohorts show that pruritus and fatigue are major factors impairing daily life [2, 24]. The PBC‐40 tool further emphasises their role as key symptoms affecting patients' well‐being [25].

Regional variations in symptom burden underscore the need for geographically tailored management strategies, as symptom severity increases with age in Italy and Japan but decreases in the UK and Spain [26]. Emerging therapies, such as IBAT inhibitors like linerixibat, show promise in reducing pruritus severity, though further studies are needed to establish their long‐term safety and efficacy [27]. These findings highlight the need for individualised, symptom‐focused strategies that address disease progression and patient‐specific factors.

This study has limitations that should be acknowledged. Firstly, selection bias may have influenced the findings, as the reliance on Italian experts could limit the generalisability of the results to other healthcare systems and populations. Secondly, response bias is possible, as clinicians with a particular interest in PBC or specific treatment philosophies may have been more inclined to participate actively in the Delphi process. Lastly, cultural and institutional biases stemming from regional variations in healthcare systems might affect the applicability of certain recommendations, particularly in countries with differing diagnostic resources, treatment accessibility, or follow‐up protocols.

Future research should focus on conducting international Delphi rounds to achieve broader consensus and account for variations in healthcare systems and practices worldwide. Additionally, controlled studies are needed to evaluate the efficacy and cost‐effectiveness of genetic testing in routine PBC management. Such studies should stratify patients based on disease progression rates and demographic factors to identify subgroups that would benefit most from these analyses. Expanding the evidence base through multicentric and globally representative research will help refine recommendations and ensure their applicability across diverse clinical settings. The Delphi survey did not explicitly evaluate symptom assessment tools (e.g., PBC‐40) or quality‐of‐life metrics. Future studies should integrate patient‐reported outcomes to align therapeutic strategies with symptom burden. Future iterations of this consensus should involve multinational panels to account for global differences in healthcare systems, diagnostic resources, and therapeutic accessibility. This would strengthen the generalisability of the proposed quality measures.

## 5 Conclusions

The Delphi Consensus Project successfully established a comprehensive agreement on the diagnosis, management, and treatment pathways for PBC. It confirms the importance of key biochemical tools, particularly non‐invasive diagnostic and monitoring procedures like FibroScan. The consensus strongly emphasises the relevance and appropriateness of available therapies, including UDCA as the first‐line treatment and OCA as an essential second‐line option for patients with inadequate responses to UDCA or UDCA‐intolerant. Specific recommendations on the early initiation of OCA in cases of UDCA intolerance or partial response further highlight its critical role in optimising disease management. The rapid development of novel PPAR agonists (e.g., elafibranor, seladelpar) underscores the need for dynamic consensus updates. Future iterations should evaluate their role in therapeutic algorithms, particularly for UDCA non‐responders.

However, the Delphi findings also reveal areas of ongoing debate. Key uncertainties remain regarding the role of genetic testing in routine practice and the necessity of liver biopsy for accurate staging of fibrosis. In particular, there is a pressing need for long‐term studies to evaluate the accuracy of VCTE in monitoring advanced fibrosis in PBC. A multicentric study comparing the outcomes of VCTE and liver biopsy across diverse PBC populations could provide valuable insights, helping to define the optimal role of these diagnostic tools in different clinical settings.

These unresolved issues reflect the complexity of PBC management and underscore the importance of evidence‐based, individualised treatment approaches. As we move forward, targeted studies and continued expert collaboration will be essential to address these knowledge gaps, ultimately paving the way for more precise and effective strategies for the diagnosis and treatment of PBC.

## Author Contributions

Study conception and design: A.C., P.P. Collection and interpretation of data: D.A., V.C., M.C., N.C., A.F., P.I., M.M., P.P., G.P., P.T., U.V.G., A.C. Statistical analysis: P.P. Manuscript drafting: A.C., G.P., P.P. Manuscript editing: D.A., V.C., M.C., N.C., A.F., P.I., M.M., P.P., G.P., P.T., U.V.G., A.C. Approval to submit: all authors.

## Ethics Statement

The authors have nothing to report.

## Conflicts of Interest

D.A.: Research Grant from Advanz Pharma. M.C.: advisor or speaker bureau member for Ipsen, Advanz, Intercept, Cymabay, Gilead, Mayoly, Echosens, Falk, Mirum, Kowa, Zydus. P.I.: Expert board member for Gilead, Ipsen, Zydus, Advanz, Barinthus, Calliditas, GSK. M.M.: Advisor for Advanz, Ipsen, Glaxo, Gilead. G.P.: speaker and/or advisor for Echosens, Novonordisk. V.C., A.C., N.C., A.F., P.P., P.T., U.V.G.: declare no conflicts of interest.

## Supporting information


**Data S1.**List of contributors.

**Data S2.**Questionnaires.

## Data Availability

The data that support the findings of this study are available from the corresponding author upon reasonable request.

## References

[1] C. Selmi and M. E. Gershwin , “Diagnosis and Classification of Reactive Arthritis,” Autoimmunity Reviews 13 (2014): 546–549, 10.1016/j.autrev.2014.01.005. 24418301

[2] A. Floreani , M. Scaffidi , B. Coco , et al., “Primary Biliary Cholangitis: Perception and Expectation of Illness,” Digestive and Liver Disease: Official Journal of the Italian Society of Gastroenterology and the Italian Association for the Study of the Liver 54, no. 9 (2022): 1230–1233, 10.1016/j.dld.2022.02.006. 35277351

[3] C. Levy , M. Manns , and G. Hirschfield , “New Treatment Paradigms in Primary Biliary Cholangitis,” Clinical Gastroenterology and Hepatology: The Official Clinical Practice Journal of the American Gastroenterological Association 21 (2023): 2076–2087, 10.1016/j.cgh.2023.02.005. 36809835

[4] K. Boonstra , U. Beuers , and C. Y. Ponsioen , “Epidemiology of Primary Sclerosing Cholangitis and Primary Biliary Cirrhosis: A Systematic Review,” Journal of Hepatology 56 (2012): 1181–1188, 10.1016/j.jhep.2011.10.025. 22245904

[5] W. J. Lammers , G. M. Hirschfield , C. Corpechot , et al., “Development and Validation of a Scoring System to Predict Outcomes of Patients With Primary Biliary Cirrhosis Receiving Ursodeoxycholic Acid Therapy,” Gastroenterology 149 (2015): 1804–1812, 10.1053/j.gastro.2015.07.061. 26261009

[6] J. Trivella , B. V. John , and C. Levy , “Primary Biliary Cholangitis: Epidemiology, Prognosis, and Treatment,” Hepatology Communications 7 (2023): e0179, 10.1097/HC9.0000000000000179. 37267215 PMC10241503

[7] I. R. Diamond , R. C. Grant , B. M. Feldman , et al., “Defining Consensus: A Systematic Review Recommends Methodologic Criteria for Reporting of Delphi Studies,” Journal of Clinical Epidemiology 67 (2014): 401–409, 10.1016/j.jclinepi.2013.12.002. 24581294

[8] H. A. Linstone , The Delphi Method: Techniques and Applications (Addison‐Wesley, 1979).

[9] J. Jones and D. Hunter , “Consensus Methods for Medical and Health Services Research,” BMJ (Clinical Research Ed.) 311 (1995): 376–380, 10.1136/bmj.311.7001.376. PMC2550437 7640549

[10] G. Rowe and G. Wright , “The Delphi Technique as a Forecasting Tool: Issues and Analysis,” International Journal of Forecasting 15 (1999): 353–375, 10.1016/S0169-2070(99)00018-7.

[11] B. Graham , G. Regehr , and J. G. Wright , “Delphi as a Method to Establish Consensus for Diagnostic Criteria,” Journal of Clinical Epidemiology 56, no. 12 (2003): 1150–1156, 10.1016/s0895-4356(03)00211-7. 14680664

[12] K. Fitch , S. J. Bernstein , M. D. Aguilar , et al., The Rand/UCLA Appropriateness Method User's Manual (Rand, 2001).

[13] A. Parés , A. Albillos , R. J. Andrade , et al., “Primary Biliary Cholangitis in Spain. Results of a Delphi Study of Epidemiology, Diagnosis, Follow‐Up and Treatment,” Revista Española de Enfermedades Digestivas 110 (2018): 641–649, 10.17235/reed.2018.5665/2018. 30032637

[14] Á. Díaz‐González , N. Fontanillas , E. Gil‐Hernández , et al., “Recomendaciones y criterios de calidad para mejorar el diagnóstico precoz de la colangitis biliar primaria,” Gastroenterología y Hepatología 47 (2024): 834–844, 10.1016/j.gastrohep.2023.12.002. 38109994

[15] R. Tomaiuolo and G. Banfi , “From Volume to Value: A Watershed Moment for the Clinical Laboratory,” Clinical Chemistry and Laboratory Medicine 62 (2024): 593–596, 10.1515/cclm-2023-0870. 37775150

[16] C. F. Murillo Perez , H. Fisher , S. Hiu , et al., “Greater Transplant‐Free Survival in Patients Receiving Obeticholic Acid for Primary Biliary Cholangitis in a Clinical Trial Setting Compared to Real‐World External Controls,” Gastroenterology 163 (2022): 1630–1642, 10.1053/j.gastro.2022.08.054. 36150526

[17] D. D'Amato , A. de Vincentis , F. Malinverno , et al., “Real‐World Experience With Obeticholic Acid in Patients With Primary Biliary Cholangitis,” JHEP Reports: Innovation in Hepatology 3 (2021): 100248, 10.1016/j.jhepr.2021.100248. 33681748 PMC7930359

[18] F. Terracciani , A. de Vincentis , D. D'Amato , et al., “Longer Transplant‐Free and Liver‐Related Event‐Free Survival in Obeticholic Acid‐Treated Patients With Primary Biliary Cholangitis Compared to External Controls From Two Large Real‐World Cohorts,” Digestive and Liver Disease 56 (2024): S3–S5, 10.1016/j.dld.2024.01.007.

[19] K. V. Kowdley , G. M. Hirschfield , C. Coombs , et al., “COBALT: A Confirmatory Trial of Obeticholic Acid in Primary Biliary Cholangitis With Placebo and External Controls,” American Journal of Gastroenterology 120, no. 2 (2024): 390–400, 10.14309/ajg.0000000000003029. 39140490 PMC11774195

[20] M. A. Brookhart , T. J. Mayne , C. Coombs , et al., “Hepatic Real‐World Outcomes With Obeticholic Acid in Primary Biliary Cholangitis (HEROES): A Trial Emulation Study Design,” Hepatology (Baltimore, Md.) (2024), 10.1097/HEP.0000000000001174. PMC12077331 39630028

[21] C. Corpechot , O. Chazouillères , A. Rousseau , et al., “A Placebo‐Controlled Trial of Bezafibrate in Primary Biliary Cholangitis,” New England Journal of Medicine 378, no. 23 (2018): 2171–2181, 10.1056/NEJMoa1714519. 29874528

[22] A. Floreani , D. Gabbia , and S. de Martin , “Obeticholic Acid for Primary Biliary Cholangitis,” Biomedicine 10 (2022): 2464, 10.3390/biomedicines10102464. PMC9599277 36289726

[23] M. J. Mayo , E. Carey , H. T. Smith , et al., “Impact of Pruritus on Quality of Life and Current Treatment Patterns in Patients With Primary Biliary Cholangitis,” Digestive Diseases and Sciences 68 (2023): 995–1005, 10.1007/s10620-022-07581-x. 35704252 PMC10406656

[24] M. Yagi , A. Tanaka , M. Abe , et al., “Symptoms and Health‐Related Quality of Life in Japanese Patients With Primary Biliary Cholangitis,” Scientific Reports 8 (2018): 12542, 10.1038/s41598-018-31063-8. 30135523 PMC6105590

[25] A. Jacoby , A. Rannard , D. Buck , et al., “Development, Validation, and Evaluation of the PBC‐40, a Disease Specific Health Related Quality of Life Measure for Primary Biliary Cirrhosis,” Gut 54 (2005): 1622–1629, 10.1136/gut.2005.065862. 15961522 PMC1774759

[26] L. Montali , A. Gragnano , M. Miglioretti , et al., “Quality of Life in Patients With Primary Biliary Cholangitis: A Cross‐Geographical Comparison,” Journal of Translational Autoimmunity 4 (2021): 100081, 10.1016/j.jtauto.2021.100081. 33554101 PMC7843515

[27] H. T. Smith , A. R. de Souza , A. H. Thompson , et al., “Cholestatic Pruritus Treatments in Primary Biliary Cholangitis and Primary Sclerosing Cholangitis: A Systematic Literature Review,” Digestive Diseases and Sciences 68 (2023): 2710–2730, 10.1007/s10620-023-07862-z. 36933112 PMC10024020

