# Supplementary material for: Quality Measures to Enhance the Management and Treatment of Primary Biliary Cholangitis: A Delphi Consensus Study
Source: Liver Int. 2025 May 2;45(6):e70118. doi: 10.1111/liv.70118 (PMC12047059; doi:10.1111/liv.70118)
Supplement: Supplementary file 1 — Data S1.List of contributors. [file LIV-45-0-s001.docx]

**Consensus-Driven Quality Measures to Enhance the Management and Treatment**

**of Primary Biliary Cholangitis: A Delphi Study**

**Supplementary Material – List of Contributors**

Ludovico Abenavoli, AOU Mater Domini - A, Catanzaro

Giuliano Alagna, USD Epatologia, AOU Sassari, Sassari

Giuseppe Alaimo, ASP di Agrigento, Agrigento

Francesco Azzaroli, Policlinico S. Orsola-Malpighi, UO Gastroenterologia, Bologna

Leonardo Baiocchi, Università di Roma Tor Vergata, Roma

Anna Baragiotta, AO Santa Maria degli Angeli, Pordenone

Francesco Bellanti, AOU Ospedali Riuniti di Foggia, Foggia

Gaetano Bertino, AOUP Vittorio Emanuele, Catania

Roberto Bertoni, AOU di Parma, Parma

Mariarosa Biagini, Università degli Studi di Firenze, Firenze

Maria Consiglia Bragazzi, Sapienza Università di Roma, Roma

Stefano Brillanti, Azienda Ospedaliera Senese, Siena

Fabrizio Bronte, AO Villa Sofia-Cervello, Palermo

Maurizia Brunetto, Policlinico Universitario di Pisa, Pisa

Mariarita Cannavò, ARNAS Garibaldi-Nesima, Catania

Benedetto Caroleo, AOU Mater Domini - B, Catanzaro

Antonino Castellaneta, Gastroenterology Unit, Policlinico di Bari Hospital, Bari

Ilaria Cavalli, ASST di Cremona, Cremona

Federica Cerini, Ospedale San Giuseppe - IRCCS Multimedica, Milano

Luchino Chessa, Liver Unit, University Hospital of Cagliari, Cagliari

Barbara Coco, Policlinico Universitario di Pisa, Unità di Epatologia, Pisa

Antonio Colecchia, Policlinico di Modena, Modena

Silvia Colombo, ASST di Bergamo Ovest, Treviglio

Maria Conti, Liver Unit, University Hospital of Cagliari, Cagliari

Raffaele Cozzolongo, Gastroenterology Unit, National Institute of Gastroenterology “S. de Bellis” Reseach Hospital, Castellana Grotte (Bari)

Giuseppe Cuccorese, Internal Medicine Ospedale “R. Dimiccoli”, Barletta

Armando Curto, AOU Careggi, Firenze

Nadia Dal Bo’, Ospedale Regionale Ca’ Foncello, Treviso

Rosanna De Marco, AO di Cosenza, Cosenza

Elisabetta Degasperi, Foundation IRCCS Ca’ Granda Ospedale Maggiore Policlinico - Center for Liver Disease, Milan

Antonio Di Giacomo, ASP di Ragusa, Ragusa

Sebastiano Di Salvo, UOC di Epatologia del Policlinico AO Mater Domini di Catanzaro, Catanzaro

Luca Fabris, AO di Padova - A, Padova

Stefano Fagiuoli, ASST Papa Giovanni XXIII, Bergamo

Elisabetta Falbo, ASP - PO di Lamezia Terme, Lamezia Terme

Alessandro Federico, Università della Campania Luigi Vanvitelli di Napoli, Napoli

Alberto Ferrarese, AOU Integrata di Verona, Verona

Francesco Foschi, Medicina Interna - Faenza, Faenza

Andrea Galli, Dipartimento di Scienze Biochimiche Sperimentali e Cliniche, Università degli Studi di Firenze, Firenze

Edoardo Giovanni Giannini, Ospedale San Martino, Genova

Lydia Giannitrapani, AOUP Paolo Giaccone, Palermo

Alessandro Granito, IRCCS AOU di Bologna, Bologna

Alberto Grassi, Medicina, Cattolica

Silvia Grazioli, Ospedale Santa Maria Degli Angeli di Pordenone, Pordenone

Donatella Ieluzzi, AOU di Verona - Borgo Roma, Verona

Agata Teresa Tiziana La Masa, ASM di Matera, Matera

Pierenrico Lecis, Ospedale San Bassiano, Bassano del Grappa

Anna Licata, AOUP Paolo Giaccone, Palermo

Ana Lleo De Nalda, Medicina Interna ed Epatologia, Humanitas Clinical and Research Center IRCCS, Milano

Elisabetta Lorefice, Ospedale San Giuseppe, Empoli

Massimiliano Loreno, ASP di Siracusa, Siracusa

Erica Nicola Lynch, AOU Careggi - A, Firenze

Salvo Madonia, AO Villa Sofia-Cervello, Palermo

Giuseppe Malizia, AO Villa Sofia-Cervello, Palermo

Massimo Marignani, Department of Liver Diseases Section, AOU Sant’Andrea Hospital, Roma

Flora Masutti, SC (UCO) Clinica Patologie del Fegato, Trieste

Andrea Mega, Azienda Sanitaria dell’Alto Adige, Bolzano

Vincenzo Messina, AO S. Anna e S. Sebastiano di Caserta, Caserta

Maria Giovanna Minissale, Ospedale Buccheri La Ferla, Palermo

Maria Cristina Morelli, Policlinico di Sant’Orsola, Bologna

Olivia Morelli, Clinica di Gastroenterologia ed Epatologia, Università degli Studi di Perugia, Perugia

Alessandra Moretti, UOC di Gastroenterologia dell’Ospedale San Filippo Neri, Roma

Filomena Morisco, Policlinico Universitario, Università Federico II, Napoli

Luigi Muratori, DIMEC Università di Bologna, Policlinico di Sant’Orsola, Bologna

Lucia Napoli, Presidio Ospedaliero di Faenza, Faenza

Grazia Niro, Ospedale Casa Sollievo della Sofferenza - A, San Giovanni Rotondo

Stefano Okolicsanyi, Azienda USL Valle d’Aosta, Aosta

Alessio Ortolani, Unità Operativa di Gastroenterologia ed Endoscopia Digestiva, AST Pesaro Urbino, Pesaro

Valeria Pace Palitti, Hepatology Unit, Santo Spirito Hospital, Pescara

Marcello Persico, UOC Clinica Medica, Medicina Interna ed Epatologia, AOU San Giovanni di Dio e Ruggi D’Aragona, Plesso “Ruggi”, Salerno

Guido Poggi, Istituto Clinico Città di Pavia - Gruppo San Donato, Pavia

Paolo Poisa, Spedali Civili di Brescia, Brescia

Francesca Ponziani, Internal Medicine and Hepatology Unit, Policlinico Gemelli, Sapienza University, Rome

Lorenzo Ridola, Sapienza Università di Roma, Roma

Cristina Rigamonti, AOU Maggiore della Carità, Novara

Roberto Risicato, ASP di Siracusa, Siracusa

Alba Rocco, Dipartimento di Medicina e Chirurgia, Università Federico II, Napoli

Maurizio Russello, ARNAS Garibaldi-Nesima, Catania

Carlo Saitta, AUOP G Martino, Messina

Mauro Sapienza, ASP di Enna, Enna

Ignazio Scalisi, ASP di Trapani, Trapani

Loredana Simone, AOU di Ferrara, Ferrara

Cinzia Simoni, ASST Valle Olona - Presidio di Gallarate, Gallarate

Lorenzo Antonio Surace, Centro Medicina del Viaggiatore e delle Migrazioni, Lamezia Terme

Natalia Terreni, Ospedale Valduce, Como

Ester Vanni, AOU Città della Salute e della Scienza di Torino, Torino

Rosanna Venere, Sapienza Università di Roma, Roma

Giovanni Vettori, Azienda per i Servizi Sanitari Provincia Autonoma di Trento, Trento

Mauro Viganò, ASST Ospedale Papa Giovanni XXIII, Bergamo

Raffaella Viganò, Ospedale Niguarda, Milano

Giovanni Vitale, IRCCS AOU Policlinico Sant’Orsola di Bologna, Bologna

Monica Zoeschg, Azienda Sanitaria dell’Alto Adige, Bolzano

Teresa Zolfino, Department of Gastroenterology, Brotzu Hospital, Cagliari
