# Supplementary material for: Quality Measures to Enhance the Management and Treatment of Primary Biliary Cholangitis: A Delphi Consensus Study
Source: Liver Int. 2025 May 2;45(6):e70118. doi: 10.1111/liv.70118 (PMC12047059; doi:10.1111/liv.70118)
Supplement: Supplementary file 2 — Data S2.Questionnaires. [file LIV-45-0-s002.docx]

**Consensus-Driven Quality Measures to Enhance the Management and Treatment**

**of Primary Biliary Cholangitis: A Delphi Study**

**Supplementary Material – Questionnaires**

**QUESTIONNAIRE 1**

a. Name / Surname: ........................

b. Year of birth: ........................

c. Year of Graduation: ........................

d. Specialisation: ........................

e. Organisation where you work: ........................

The organisation where you work is [you can select multiple answers]

1. ☐ University

2. ☐ Public hospital

3. ☐ Private hospital

4. ☐ Territorial service (outpatient clinic)

f. Main area of activity: ........................

g. For how many years have you been involved in your main area of activity: ........................

h. Your role in the organisation: ........................

i. Do you belong to one or more scientific societies? Yes/No If yes, which ones?

j. Have you published in the last 10 years scientific contributions in journals listed on PubMed on the topic of PBC? Yes/No

**GENERAL FRAMEWORK**

1. Does your centre have a device for measuring liver stiffness using VCTE (i.e. FibroScan)?

a. Yes

b. No

c. No, but it is possible to use it at a nearby centre

2. Is it possible to perform a liver biopsy at your centre?

a. Yes

b. No

c. No, but it is possible to perform it at a nearby centre

3. Does your centre have a radiology service where MRI can be performed?

a. Yes

b. No

c. No, but it is possible to use this service at a nearby centre

4. Is there a laboratory in your centre capable of performing investigations such as virological screening and autoimmunity studies?

a. Yes

b. No

c. No, but it is possible to use this service at a nearby centre

5. Does your centre have an endoscopy service with the possibility of performing Ecoendoscopy?

a. Yes

b. No

c. No, but it is possible to use this service at a nearby centre

6. Do you have direct contact with colleagues at a centre with specific clinical and research experience on PBC or experts in PBC management?

a. Yes, I work in a centre with specific clinical and research experience on PBC

b. Yes, I have formal contact with a centre with specific clinical and research experience on PBC

c. Yes, I have informal contact with a centre with specific clinical and research experience on PBC

d. No, no contact with a centre with specific clinical and research experience on PBC

**PATIENTS JOURNEY**

1. In your experience, where is the diagnosis of PBC most frequently made?

a. In your centre/clinic 1 2 3 4 5 6 7 8 9

b. In a centre with specific clinical and research experience in PBC 1 2 3 4 5 6 7 8 9

2. With reference to the PBC patients you usually follow, from where does the referral take place?

a. Direct access 1 2 3 4 5 6 7 8 9

b. General practitioner 1 2 3 4 5 6 7 8 9

c. Gastroenterology specialist 1 2 3 4 5 6 7 8 9

d. Other specialists 1 2 3 4 5 6 7 8 9

3. If the patient had direct access, which method did you use?

a. Online search 1 2 3 4 5 6 7 8 9

b. Social platforms 1 2 3 4 5 6 7 8 9

c. Advice/suggestion from third parties (relatives, friends, acquaintances, other patients...) 1 2 3 4 5 6 7 8 9

d. Use of artificial intelligence (i.e. ChatGPT) 1 2 3 4 5 6 7 8 9

4. How long does the average time elapse between the manifestation of symptoms and the diagnosis/staging of the disease?

a. Less than 3 months 1 2 3 4 5 6 7 8 9

b. 3 to 6 months 1 2 3 4 5 6 7 8 9

c. More than 6 months 1 2 3 4 5 6 7 8 9

**PATIENT IDENTIFICATION, DIAGNOSIS AND STAGING**

1. ALP alteration in asymptomatic patients without concomitant diseases requires:

IDd01_r1 - No further investigation 1 2 3 4 5 6 7 8 9

IDd01_r2 - Medical and pharmacological history 1 2 3 4 5 6 7 8 9

IDd01_r3 - Biochemical study of liver function 1 2 3 4 5 6 7 8 9

IDd01_r4 - Virological and autoimmunity screening 1 2 3 4 5 6 7 8 9

IDd01_r5 - Abdominal ultrasound 1 2 3 4 5 6 7 8 9

IDd01_r6 - Biochemical monitoring every 3-6 months 1 2 3 4 5 6 7 8 9

2. The alteration of ALP in a patient with itching and fatigue, without any concomitant conditions, requires:

IDd02_r1 - No further investigation 1 2 3 4 5 6 7 8 9

IDd02_r2 - Medical and pharmacological history 1 2 3 4 5 6 7 8 9

IDd02_r3 - Biochemical study of liver function 1 2 3 4 5 6 7 8 9

IDd02_r4 - Virological and autoimmunity screening 1 2 3 4 5 6 7 8 9

IDd02_r5 - Abdominal ultrasound 1 2 3 4 5 6 7 8 9

IDd02_r6 - Biochemical monitoring every 3-6 months 1 2 3 4 5 6 7 8 9

3. The alteration of ALP in an asymptomatic patient with concomitant pathological conditions (Hashimoto's thyroiditis, Sjögren's syndrome, celiac disease, or systemic sclerosis) requires:

IDd03_r1 - No further investigation 1 2 3 4 5 6 7 8 9

IDd03_r2 - Medical and pharmacological history 1 2 3 4 5 6 7 8 9

IDd03_r3 - Biochemical study of liver function 1 2 3 4 5 6 7 8 9

IDd03_r4 - Virological and autoimmunity screening 1 2 3 4 5 6 7 8 9

IDd03_r5 - Abdominal ultrasound 1 2 3 4 5 6 7 8 9

IDd03_r6 - Biochemical monitoring every 3-6 months 1 2 3 4 5 6 7 8 9

4. The alteration of ALP associated with itching and fatigue in the presence of concomitant pathological conditions (Hashimoto's thyroiditis, Sjögren's syndrome, celiac disease, or systemic sclerosis) requires:

IDd04_r1 - No further investigation 1 2 3 4 5 6 7 8 9

IDd04_r2 - Medical and pharmacological history 1 2 3 4 5 6 7 8 9

IDd04_r3 - Biochemical study of liver function 1 2 3 4 5 6 7 8 9

IDd04_r4 - Virological and autoimmunity screening 1 2 3 4 5 6 7 8 9

IDd04_r5 - Abdominal ultrasound 1 2 3 4 5 6 7 8 9

IDd04_r6 - Biochemical monitoring every 3-6 months 1 2 3 4 5 6 7 8 9

5. The evidence of chronic cholestasis (elevated ALP and GGT and/or bilirubin > 6 months), regardless of symptoms, initially requires:

IDd05_r1 - Pathological history, remote and pharmacological 1 2 3 4 5 6 7 8 9

IDd05_r2 - Virological screening and autoimmunity 1 2 3 4 5 6 7 8 9

IDd05_r3 - Ultrasound of the abdomen 1 2 3 4 5 6 7 8 9

IDd05_r4 - Measurement of liver stiffness by VCTE 1 2 3 4 5 6 7 8 9

6. The evidence of chronic cholestasis (elevated ALP and GGT and/or bilirubin > 6 months), regardless of symptoms, when first-level tests previously listed are inconclusive, requires:

IDd06_r1 - MRCP +/- EUS 1 2 3 4 5 6 7 8 9

IDd06_r2 - Liver biopsy 1 2 3 4 5 6 7 8 9

IDd06_r3 - Genetic study (i.e. ATP8B1, ABCB11, ABCB4) 1 2 3 4 5 6 7 8 9

7. The detection of AMA+ in the absence of chronic cholestasis (elevated ALP and GGT and/or bilirubin > 6 months), regardless of symptoms, initially requires:

IDd07_r1 - Search for other concomitant autoimmune diseases 1 2 3 4 5 6 7 8 9

IDd07_r2 - Ultrasound of the abdomen 1 2 3 4 5 6 7 8 9

IDd07_r3 - Measurement of liver stiffness by VCTE 1 2 3 4 5 6 7 8 9

IDd07_r4 - Annual biohumoral monitoring 1 2 3 4 5 6 7 8 9

8. The detection of AMA+ in the absence of chronic cholestasis (elevated ALP and GGT and/or bilirubin > 6 months), regardless of symptoms, when the previously listed first-level tests are inconclusive, requires:

IDd08_r1 - MRCP +/- EUS 1 2 3 4 5 6 7 8 9

IDd08_r2 - Liver biopsy 1 2 3 4 5 6 7 8 9

IDd08_r3 - Genetic study (i.e. ATP8B1, ABCB11, ABCB4) 1 2 3 4 5 6 7 8 9

9. The detection of AMA+ in the presence of chronic cholestasis (elevated ALP and GGT and/or bilirubin > 6 months), regardless of symptoms, initially requires:

IDd09_r1 - Search for other concomitant autoimmune diseases 1 2 3 4 5 6 7 8 9

IDd09_r2 - Ultrasound of the abdomen 1 2 3 4 5 6 7 8 9

IDd09_r3 - Measurement of liver stiffness by VCTE 1 2 3 4 5 6 7 8 9

10. The detection of AMA+ in the presence of chronic cholestasis (elevated ALP and GGT and/or bilirubin > 6 months), regardless of symptoms, when the previously listed first-level tests are inconclusive, requires:

IDd10_r1 - MRCP +/- EUS 1 2 3 4 5 6 7 8 9

IDd10_r2 - Liver biopsy 1 2 3 4 5 6 7 8 9

IDd10_r3 - Genetic study (i.e. ATP8B1, ABCB11, ABCB4) 1 2 3 4 5 6 7 8 9

11. The detection of ANA+ (anti-sp100, anti-gp210, anti-centromere) in the absence of chronic cholestasis (elevated ALP and GGT and/or bilirubin > 6 months), regardless of symptoms, initially requires:

IDd11_r1 - Screening for other concomitant autoimmune diseases 1 2 3 4 5 6 7 8 9

IDd11_r2 - Abdominal ultrasound 1 2 3 4 5 6 7 8 9

IDd11_r3 - Measurement of liver stiffness via VCTE 1 2 3 4 5 6 7 8 9

IDd11_r4 - Annual biomarker monitoring 1 2 3 4 5 6 7 8 9

12. The detection of ANA+ (anti-sp100, anti-gp210, anti-centromere) in the absence of chronic cholestasis (elevated ALP and GGT and/or bilirubin > 6 months), regardless of symptoms, when the previously listed first-level tests are inconclusive, requires:

IDd12_r1 - MRCP +/- EUS 1 2 3 4 5 6 7 8 9

IDd12_r2 - Liver biopsy 1 2 3 4 5 6 7 8 9

IDd12_r3 - Genetic testing (i.e., ATP8B1, ABCB11, ABCB4) 1 2 3 4 5 6 7 8 9

13. The detection of ANA+ (anti-sp100, anti-gp210, anti-centromere) in the presence of chronic cholestasis (elevated ALP and GGT and/or bilirubin > 6 months), regardless of symptoms, requires:

IDd13_r1 - Screening for other concomitant autoimmune diseases 1 2 3 4 5 6 7 8 9

IDd13_r2 - Abdominal ultrasound 1 2 3 4 5 6 7 8 9

IDd13_r3 - Measurement of liver stiffness via VCTE 1 2 3 4 5 6 7 8 9

IDd13_r4 - MRCP +/- EUS 1 2 3 4 5 6 7 8 9

IDd13_r5 - Liver biopsy 1 2 3 4 5 6 7 8 9

14. The detection of ANA+ (anti-sp100, anti-gp210, anti-centromere) in the presence of chronic cholestasis (elevation of ALP and GGT and/or bilirubin > 6 months), irrespective of symptomatology, where the first-level investigations listed above have not been conclusive, requires:

IDd14_r1 - MRCP +/- EUS 1 2 3 4 5 6 7 8 9

IDd14_r2 - Liver biopsy 1 2 3 4 5 6 7 8 9

IDd14_r3 - Genetic testing (i.e., ATP8B1, ABCB11, ABCB4) 1 2 3 4 5 6 7 8 9

15. The detection of chronic cholestasis (elevated ALP and GGT and/or bilirubin > 6 months) in the presence of AMA/ANA, regardless of symptoms, requires:

IDd15_r1 - Biomarker monitoring every 3-6 months 1 2 3 4 5 6 7 8 9

IDd15_r2 - Abdominal ultrasound 1 2 3 4 5 6 7 8 9

IDd15_r3 - Measurement of liver stiffness via VCTE 1 2 3 4 5 6 7 8 9

IDd15_r4 - MRCP +/- EUS 1 2 3 4 5 6 7 8 9

IDd15_r5 - Liver biopsy 1 2 3 4 5 6 7 8 9

IDd15_r6 - Re-testing for autoimmunity every 6-12 months 1 2 3 4 5 6 7 8 9

IDd15_r7 - Genetic testing (i.e., ATP8B1, ABCB11, ABCB4) 1 2 3 4 5 6 7 8 9

16. In the presence of a clinical diagnosis of PBC (chronic cholestasis and AMA+), staging of fibrosis and the potential presence of portal hypertension requires:

IDd16_r1 - Albumin and platelet dosage 1 2 3 4 5 6 7 8 9

IDd16_r2 - EGDS 1 2 3 4 5 6 7 8 9

IDd16_r3 - Abdominal ultrasound 1 2 3 4 5 6 7 8 9

IDd16_r4 - Measurement of liver stiffness via VCTE 1 2 3 4 5 6 7 8 9

IDd16_r5 - Liver biopsy 1 2 3 4 5 6 7 8 9

IDd16_r6 - Not necessary 1 2 3 4 5 6 7 8 9

17. In which of the following settings is a liver biopsy required:

IDd17_r1 - Disease staging and degree of fibrosis 1 2 3 4 5 6 7 8 9

IDd17_r2 - Cholestasis without autoantibodies 1 2 3 4 5 6 7 8 9

IDd17_r3 - Suspected ductopenic variant 1 2 3 4 5 6 7 8 9

IDd17_r4 - Overlap syndrome 1 2 3 4 5 6 7 8 9

IDd17_r5 - Coexistence of other etiologies (i.e., MASLD) 1 2 3 4 5 6 7 8 9

IDd17_r6 - Poor Response to UDCA 1 2 3 4 5 6 7 8 9

18. VCTE can be used for:

IDd18_r1 - Staging of the disease and degree of fibrosis along with biopsy 1 2 3 4 5 6 7 8 9

IDd18_r2 - Prognosis assessment 1 2 3 4 5 6 7 8 9

IDd18_r3 - Staging of the disease and degree of fibrosis instead of biopsy 1 2 3 4 5 6 7 8 9

IDd18_r4 - Only for the diagnosis of advanced fibrosis and cirrhosis 1 2 3 4 5 6 7 8 9

IDd18_r5 - Non-invasive monitoring of fibrosis 1 2 3 4 5 6 7 8 9

**TREATMENT AND FOLLOW-UP**

1. UDCA in PBC is used:

TFd01_r1 - During pregnancy and breastfeeding 1 2 3 4 5 6 7 8 9

TFd01_r2 - In a single dose 1 2 3 4 5 6 7 8 9

TFd01_r3 - In fractionated doses 1 2 3 4 5 6 7 8 9

TFd01_r4 - After main meals 1 2 3 4 5 6 7 8 9

2. The assessment of response to therapy is performed:

TFd02_r1 - Six months after starting UDCA 1 2 3 4 5 6 7 8 9

TFd02_r2 - Twelve months after starting UDCA 1 2 3 4 5 6 7 8 9

TFd02_r3 - Through measurement of ALP, GGT, bilirubin, and transaminases 1 2 3 4 5 6 7 8 9

3. Referral to a centre with specific clinical expertise and research on PBC is required if:

TFd03_r1 - Overlap syndrome 1 2 3 4 5 6 7 8 9

TFd03_r2 - No response/Partial response to UDCA 1 2 3 4 5 6 7 8 9

TFd03_r3 - Coexistence of other etiologies (i.e., MASLD) 1 2 3 4 5 6 7 8 9

TFd03_r4 - Coexistence of other associated autoimmune diseases 1 2 3 4 5 6 7 8 9

4. In the case of absent or partial response to UDCA, consideration should be given to:

TFd04_r1 - Overlap syndrome 1 2 3 4 5 6 7 8 9

TFd04_r2 - Coexistence of other etiologies (i.e., MASLD) 1 2 3 4 5 6 7 8 9

TFd04_r3 - Ductopenic variant 1 2 3 4 5 6 7 8 9

TFd04_r4 - Potential ductal reaction 1 2 3 4 5 6 7 8 9

TFd04_r5 - Diagnostic workup (i.e., MRCP +/- EUS, Liver Biopsy) at their own centre 1 2 3 4 5 6 7 8 9

TFd04_r6 - Referral to a centre with specific clinical and research expertise in PBC 1 2 3 4 5 6 7 8 9

5. At the time of PBC diagnosis and during follow-up, it is recommended:

TFd05_r1 - Annual measurement of vitamin D, calcium, and PTH 1 2 3 4 5 6 7 8 9

TFd05_r2 - DEXA every 1-4 years 1 2 3 4 5 6 7 8 9

TFd05_r3 - Screening for Sjögren's syndrome, systemic sclerosis, and celiac disease 1 2 3 4 5 6 7 8 9

TFd05_r4 - Measurement of TSH 1 2 3 4 5 6 7 8 9

TFd05_r5 - Measurement of total cholesterol 1 2 3 4 5 6 7 8 9

TFd05_r6 - Assessment of cardiovascular risk 1 2 3 4 5 6 7 8 9

TFd05_r7 - Referral to the specialist 1 2 3 4 5 6 7 8 9

6. Follow-up in the primary care setting is expected:

TFd06_r1 - Non-cirrhotic patients 1 2 3 4 5 6 7 8 9

TFd06_r2 - Asymptomatic patients 1 2 3 4 5 6 7 8 9

TFd06_r3 - Patients responsive to treatment with UDCA 1 2 3 4 5 6 7 8 9

TFd06_r4 - In all cases of PBC 1 2 3 4 5 6 7 8 9

TFd06_r5 - Normal liver tests for at least 12 months 1 2 3 4 5 6 7 8 9

7. The performance of EGDS for screening of portal hypertension in patients with PBC is recommended if:

TFd07_r1 - Histologically documented cirrhosis

TFd07_r2 - If LSM ≥ 20 kPa or PLT < 150,000/mm³ 1 2 3 4 5 6 7 8 9

TFd07_r3 - If LSM ≥ 20 kPa or PLT < 150,000/mm³ in the absence of NSBB 1 2 3 4 5 6 7 8 9

TFd07_r4 - In follow-up according to the Baveno VI criteria 1 2 3 4 5 6 7 8 9

TFd07_r5 - If ultrasound signs of portal hypertension are present 1 2 3 4 5 6 7 8 9

8. The role of obeticholic acid in PBC:

TFd08_r1 - Only as second line after UDCA 1 2 3 4 5 6 7 8 9

TFd08_r2 - In compensated cirrhosis (Child-Pugh A) 1 2 3 4 5 6 7 8 9

TFd08_r3 - Initial dosage of 5 mg up to 10 mg 1 2 3 4 5 6 7 8 9

TFd08_r4 - Requires discontinuation if side effects appear 1 2 3 4 5 6 7 8 9

TFd08_r5 - May be useful to introduce before 12 months after starting UDCA

if intolerance/insufficient response to UDCA 1 2 3 4 5 6 7 8 9

9. Given the current state of knowledge, regardless of approved indications, the use of bezafibrate in PBC can be considered:

TFd09_r1 - Always in conjunction with UDCA 1 2 3 4 5 6 7 8 9

TFd09_r2 - For the treatment of compensated cirrhosis 1 2 3 4 5 6 7 8 9

TFd09_r3 - For the reduction of mortality in PBC 1 2 3 4 5 6 7 8 9

TFd09_r4 - Requires monitoring of creatinine 1 2 3 4 5 6 7 8 9

TFd09_r5 - Use in other contexts (i.e., in Association with OCA) 1 2 3 4 5 6 7 8 9

10. Given the current state of knowledge, regardless of approved indications, the use of budesonide in PBC can be considered:

TFd10_r1 - Always in combination with UDCA 1 2 3 4 5 6 7 8 9

TFd10_r2 - Only in non-cirrhotic patients 1 2 3 4 5 6 7 8 9

TFd10_r3 - In the case of active hepatic inflammation 1 2 3 4 5 6 7 8 9

TFd10_r4 - In the presence of autoimmune hepatitis 1 2 3 4 5 6 7 8 9

TFd10_r5 - Only for non-long-term treatments 1 2 3 4 5 6 7 8 9

11. In the case of PBC in pregnant women:

TFd11_r1 - The use of UDCA is recommended throughout pregnancy 1 2 3 4 5 6 7 8 9

TFd11_r2 - Cholestyramine and rifampicin are indicated in the third trimester 1 2 3 4 5 6 7 8 9

TFd11_r3 - The use of plasma exchange is indicated if the itching is untreatable 1 2 3 4 5 6 7 8 9

TFd11_r4 - Supplementation of fat-soluble vitamins is mandatory 1 2 3 4 5 6 7 8 9

TFd11_r5 - Referral to a centre with specific clinical and research expertise in PBC is necessary 1 2 3 4 5 6 7 8 9

12. The management of mild pruritus in PBC includes:

TFd12_r1 - The use of cholestyramine as first-line 1 2 3 4 5 6 7 8 9

TFd12_r2 - The use of bezafibrate and rifampicin only if intolerance

or no response to cholestyramine 1 2 3 4 5 6 7 8 9

TFd12_r3 - The management by the primary care physician 1 2 3 4 5 6 7 8 9

13. The management of moderate-severe pruritus in PBC includes:

TFd13_r1 - The use of cholestyramine as first-line 1 2 3 4 5 6 7 8 9

TFd13_r2 - The use of bezafibrate in all cases except decompensated cirrhosis 1 2 3 4 5 6 7 8 9

TFd13_r3 - The use of rifampicin in cases of intolerance or non-response to bezafibrate 1 2 3 4 5 6 7 8 9

TFd13_r4 - The use of naltrexone or sertraline in cases of intolerance or contraindications

to both bezafibrate and rifampicin 1 2 3 4 5 6 7 8 9

TFd13_r5 - Referral to a centre with specific clinical and research experience in PBC

and OLT evaluation 1 2 3 4 5 6 7 8 9

14. Referral to a centre with specific clinical expertise and research on PBC for potential liver transplantation evaluation is indicated if:

TFd14_r1 - In all cases of MELD > 15 1 2 3 4 5 6 7 8 9

TFd14_r2 - Presence of complications of liver cirrhosis, including HCC 1 2 3 4 5 6 7 8 9

TFd14_r3 - Persistently elevated total bilirubin > 3 mg/dL 1 2 3 4 5 6 7 8 9

TFd14_r4 - Severe pruritus resistant to medical therapy regardless of MELD 1 2 3 4 5 6 7 8 9

**QUESTIONNAIRE 2**

**PATIENT IDENTIFICATION, DIAGNOSIS AND STAGING**

1. Genetic testing (i.e., ATP8B1, ABCB11, ABCB4) is required:

IDd1_r1 - In the case of diagnostic uncertainty from liver biopsy 1 2 3 4 5 6 7 8 9

IDd1_r2 - In the case of diagnostic uncertainty on MRCP ± EUS 1 2 3 4 5 6 7 8 9

IDd1_r3 - In both of the above cases 1 2 3 4 5 6 7 8 9

IDd1_r4 - Regardless of the results of the previous tests in cases of strong clinical suspicion 1 2 3 4 5 6 7 8 9

IDd1_r5 - In all cases of chronic cholestasis of NDD regardless of the positivity of AMA/ANA 1 2 3 4 5 6 7 8 9

2. A liver biopsy is indicated:

IDd2_r1 - For AMA+ or ANA+ in the absence of cholestasis 1 2 3 4 5 6 7 8 9

IDd2_r2 - For AMA+ or ANA+ in the presence of chronic cholestasis as a first-line approach 1 2 3 4 5 6 7 8 9

IDd2_r3 - For AMA+ or ANA+ in the presence of chronic cholestasis in case of doubt after

other diagnostic investigations (abdominal ultrasound, VCTE, MRCP ± EUS) 1 2 3 4 5 6 7 8 9

IDd2_r4 - Always for fibrosis staging 1 2 3 4 5 6 7 8 9

3. VCTE can be used to:

IDd3_r1 - For disease staging and degree of fibrosis along with biopsy 1 2 3 4 5 6 7 8 9

IDd3_r2 - For disease staging and degree of fibrosis regardless of biopsy 1 2 3 4 5 6 7 8 9

IDd3_r3 - Only for the identification and monitoring of advanced fibrosis 1 2 3 4 5 6 7 8 9

Dd3_r4 - For fibrosis staging if AMA+ or ANA+ in the absence of chronic cholestasis 1 2 3 4 5 6 7 8 9

IDd3_r5 - For fibrosis staging if AMA+ or ANA+ in the presence of chronic cholestasis 1 2 3 4 5 6 7 8 9

**TREATMENT AND FOLLOW-UP**

1. The management by the General Practitioner in PBC is expected for:

TFd1_r1 - Non-cirrhotic patients 1 2 3 4 5 6 7 8 9

TFd1_r2 - Asymptomatic patients 1 2 3 4 5 6 7 8 9

TFd1_r3 - Patients responsive to treatment with UDCA 1 2 3 4 5 6 7 8 9

TFd1_r4 - In all cases of PBC 1 2 3 4 5 6 7 8 9

TFd1_r5 - Normal liver tests for at least 12 months 1 2 3 4 5 6 7 8 9

TFd1_r6 - Not required 1 2 3 4 5 6 7 8 9

2. Continuous evaluation and regular follow-up of the patient should be performed:

TFd2_r1 - At least every 3 months in patients with a high risk of disease progression 1 2 3 4 5 6 7 8 9

TFd2_r2 - At least every 6 months in patients with intermediate/high risk of

disease progression 1 2 3 4 5 6 7 8 9

TFd2_r3 - At least once a year in patients with low risk of disease progression 1 2 3 4 5 6 7 8 9

TFd2_r4 - 6 months after the start of UDCA therapy to schedule the potential second-line

initiation visit at 12 months 1 2 3 4 5 6 7 8 9

- - - - -
